# Hypoxia-induced activation of NDR2 underlies brain metastases from Non-Small Cell Lung Cancer

**DOI:** 10.1038/s41419-023-06345-3

**Published:** 2023-12-13

**Authors:** Jérôme Levallet, Tiphaine Biojout, Céline Bazille, Manon Douyère, Fatéméh Dubois, Dimitri Leite Ferreira, Jasmine Taylor, Sylvain Teulier, Jérôme Toutain, Nicolas Elie, Myriam Bernaudin, Samuel Valable, Emmanuel Bergot, Guénaëlle Levallet

**Affiliations:** 1 Université de Caen Normandie, CNRS, Normandie Université, ISTCT UMR6030, GIP CYCERON, Caen, F-14074 France; 2 Department of Pathology, CHU de Caen, Caen, F-14000 France; 3 Structure Fédérative D’oncogénétique cyto-MOléculaire du CHU de Caen (SF-MOCAE), CHU de Caen, Caen, F-14000 France; 4 Department of Pulmonology & Thoracic Oncology, CHU de Caen, Caen, F-14000 France; 5 CNRS, Université de Caen Normandie, Normandie Université, ISTCT UMR6030, GIP CYCERON, Caen, F-14074 France; 6 Normandie Univ, UNICAEN, Federative Structure 4207 “Normandie Oncologie”, Service Unit PLATON, Virtual’His platform, Caen, France; Normandie Univ, UNICAEN, Service Unit EMERODE, Centre de Microscopie Appliquée à la Biologie, CMABio³, Caen, France

**Keywords:** Prognostic markers, Diseases

## Abstract

The molecular mechanisms induced by hypoxia are misunderstood in non-small cell lung cancer (NSCLC), and above all the hypoxia and RASSF1A/Hippo signaling relationship. We confirmed that human NSCLC (*n* = 45) as their brain metastases (BM) counterpart are hypoxic since positive with CAIX-antibody (target gene of Hypoxia-inducible factor (HIF)). A severe and prolonged hypoxia (0.2% O2, 48 h) activated YAP (but not TAZ) in Human Bronchial Epithelial Cells (HBEC) lines by downregulating RASSF1A/kinases Hippo (except for NDR2) regardless their promoter methylation status. Subsequently, the NDR2-overactived HBEC cells exacerbated a HIF-1A, YAP and C-Jun-dependent-amoeboid migration, and mainly, support BM formation. Indeed, NDR2 is more expressed in human tumor of metastatic NSCLC than in human localized NSCLC while NDR2 silencing in HBEC lines (by shRNA) prevented the xenograft formation and growth in a lung cancer-derived BM model in mice. Collectively, our results indicated that NDR2 kinase is over-active in NSCLC by hypoxia and supports BM formation. NDR2 expression is thus a useful biomarker to predict the metastases risk in patients with NSCLC, easily measurable routinely by immunohistochemistry on tumor specimens.

## Introduction

Hypoxia (oxygen deprivation) supports genomic instability, aggressiveness of tumor cells, formation of metastases, and resistance to treatment by non-small-cell lung cancers (NSCLC) [1–3] by incompletely established mechanisms. One can hypothesize that hypoxia disrupts the RASSF1A (Ras association domain family 1 isoform A)/Hippo signaling pathway [4–9] since YAP is active in several hypoxic tumors [10] and interact either with (**i)** hypoxia-inducible factor-1α (HIF-1A) to promote pancreatic ductal adenocarcinoma invasion [9] or hepatocellular carcinoma cell glycolysis under hypoxic stress [11], or with (**ii)** HIF-2A to promote the progression of colon cancer [12]. Regarding NSCLC, that hypoxia could disrupt the RASSF1A/Hippo signaling pathway, is of particular interest since Hippo pathway is already known to be altered following the loss of expression of RASSF1A in 25% of patient with NSCLC [13], leading to aberrant activation of both the Hippo kinase, NDR2 and the Hippo effector, YAP [14] and supporting the subsequent initiation and dissemination of NSCLC [14, 15]. Only one paper reports the role of RASSF1A-HIF-1A loop, in a subset of NSCLC still expressing RASSF1A and the primary cancer cells isolated from the same tumors, independent of Hippo signaling [16]. Here, we decipher the HIF-1A/YAP/TAZ relationship in presence or absence of RASSF1A in Human Bronchial Epithelial Cells (HBEC) lines grown under severe (0.2% O_2_) and prolonged (48 h) hypoxia (i.e. as conditions present in the core/bulk of lung tumor [17–19]). We seek to determine whether NDR2 is hyperactivated early (following epigenetic dysregulation) or late (by hypoxia) during the natural history of NSCLC and could be a useful tool to diagnose metastatic tumors in view of these pro-migratory properties.

## Results

### Human primitive NSCLC as their brain metastases are hypoxic

The H-Score of carbonic anhydrase 9 (CAIX), a transcriptional target of HIF-1A [20], is similar between primary tumors of patients with localized NSCLC (62.4 ± 12.3) or metastatic NSCLC (71.2 ± 19.7), and comparable between primary tumors and brain metastasis (65.4 ± 17.1, *n* = 20) from the same patients (Fig. S1).

### Hypoxia (0.2% O_2_, 48 h) activates YAP but not TAZ in HBEC lines

HBEC-3 (Fig. S2) as the other HBEC lines (Fig. S3) survive to a hypoxia (48 h, 0.2% O_2_, confirmed by the nuclear accumulation of HIF-1A (HBEC-3: Fig. S2A, A549: S2B)) but reach confluence slowly than in normoxia (HBEC-3: Fig. S2C; A549: Fig. S2D) since then apoptosis increases (Caspase3/7 activity, HBEC-3: Fig. S2E, A549: Fig. S2F). However, HBEC-3 cells (Fig. S2G) as A549 cells (Fig. S2H) still incorporate BrdU between 24 and 48 h.

**Fig. 1:**
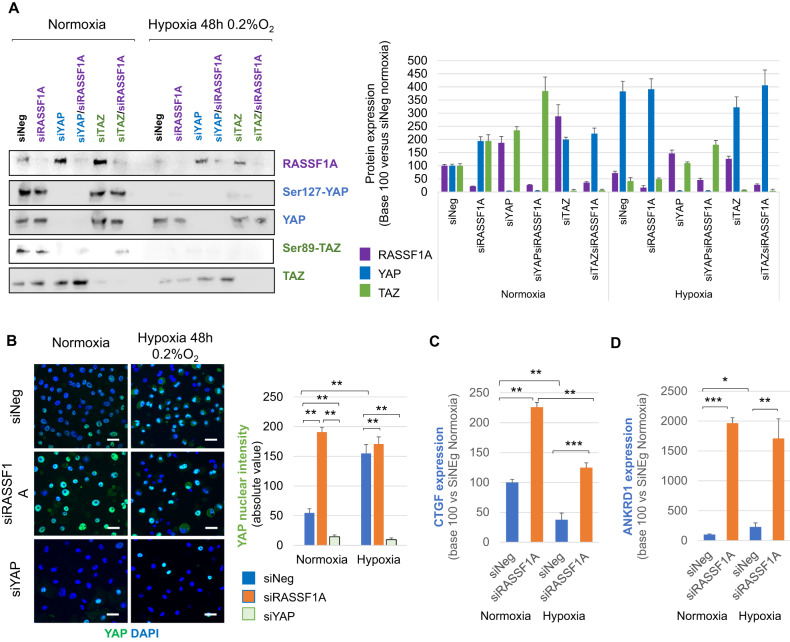
Severe and prolonged hypoxia activates YAP and downregulates TAZ in HBEC-3 cells. **A** Western blot of members of the Hippo pathway on HBEC-3, expressing or not RASSF1A, after 48 h of normoxia/hypoxia and associated quantification (right panel). **B** Immunostaining of YAP on HBEC-3 expressing or not RASSF1A and YAP, after 48 h of normoxia/hypoxia and Graphic representation of YAP nuclear intensity. **C**, **D** Representations of the mRNAs expression of the target genes of YAP: CTGF (**C**) and ANKRD1 (**D**). P-value *p < 0.05, **p < 0.01 and ***p < 0.001 (SEM *n* ≥ 3).

Hypoxia (0.2% O_2_, 48 h) increases dephosphorylated YAP protein while the expression of TAZ drastically decreases in HBEC-3 (Fig. 1A). In line, hypoxia (0.2% O_2_, 48 h) increases the YAP nuclear intensity like the RASSF1A depletion without additive effect between these two events (Fig. 1B) while TAZ nuclear signal decreases (Fig. S4). The RASSF1A depletion, which causes the nuclear translocation of active YAP in HBEC-3 cells [15], significantly increases the expression of these two target genes of YAP [21, 22] (*CTGF* (Fig. 1C) and *ANKRD1* (Fig. 1D)) confirming that YAP is active when the HBEC-3 cells are in hypoxia (0.2% O_2_, 48 h). TAZ decrease (Western-Blot: Fig. S5A, B, C, immunofluorescence: Fig. S5H) but YAP accumulation (Fig. S5A, D, E) and activation (YAP nuclear accumulation: Fig. S5F; *CTGF* and *ANKRD1* expression: Fig. S5G) were also reported in others HBEC cells grown in hypoxia (0.2% O_2_, confirmed by HIF1-A nuclear accumulation (Fig. S5I).

### Hypoxia (0.2% O_2_, 48 h) downregulated RASSF1A/kinases Hippo in HBEC lines except for NDR2

**Fig. 2:**
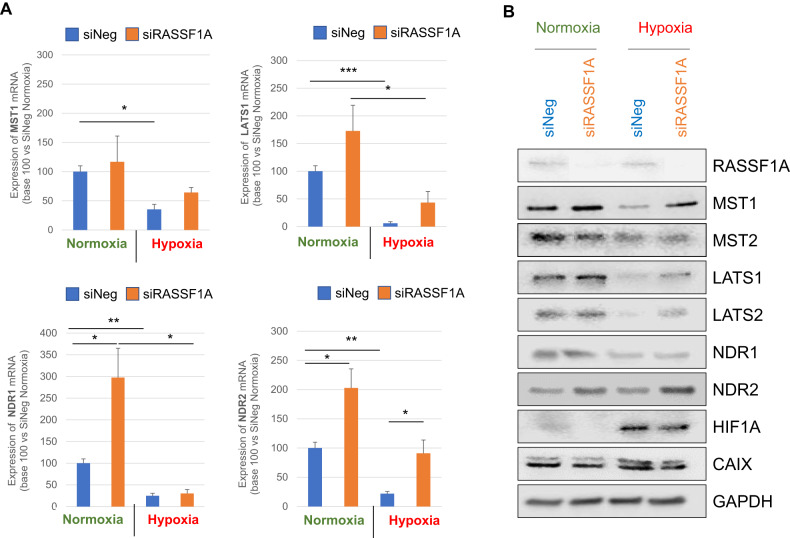
Severe and prolonged hypoxia preserves NDR2 protein expression but decreases expression of other Hippo pathway kinases in HBEC-3 cells. **A** Graphic representation of the mRNAs expression of the kinases of the Hippo pathway MST1, LATS1 and NDR1/2 (A-D) by qPCR on the HBEC-3 cells. *P*-value *p < 0.05, **p < 0.01 and ***p < 0.001 (SEM *n* ≥ 3). **B** Western blot of Hippo pathway members in HBEC-3 cells, expressing or not RASSF1A, after 48 h of normoxia / hypoxia (representative experiment).

Hypoxia (0.2% O_2_ for 48 h) decreases the expression of YAP’s kinases MST1, LATS1, and NDR1/2 mRNA in RASSF1-depleted or not HBEC cells with (Fig. 2A), however, at protein level, NDR2 is preserved (Fig. 2B). In line, NDR2 kinase expression is not significantly modified by hypoxia, whatever the cell line considered (Fig. S6A, E). Hypoxia (0.2% O_2_, 48 h) decreases Hippo kinases, except NDR2, without changing the methylation status of the promoters of their genes or of the target gene of YAP, *ANKRD1*, known to be inactivated/hypermethylated in NSCLC [8, 22] (HBEC-3: Fig. S7A, A549: Fig. S7B).

### Hypoxia (0.2% O2, 48 h) exacerbates the ability of NDR2-overactived NSCLC cells to perform a YAP/C-Jun and HIF-1A-dependent amoeboid migration

**Fig. 3:**
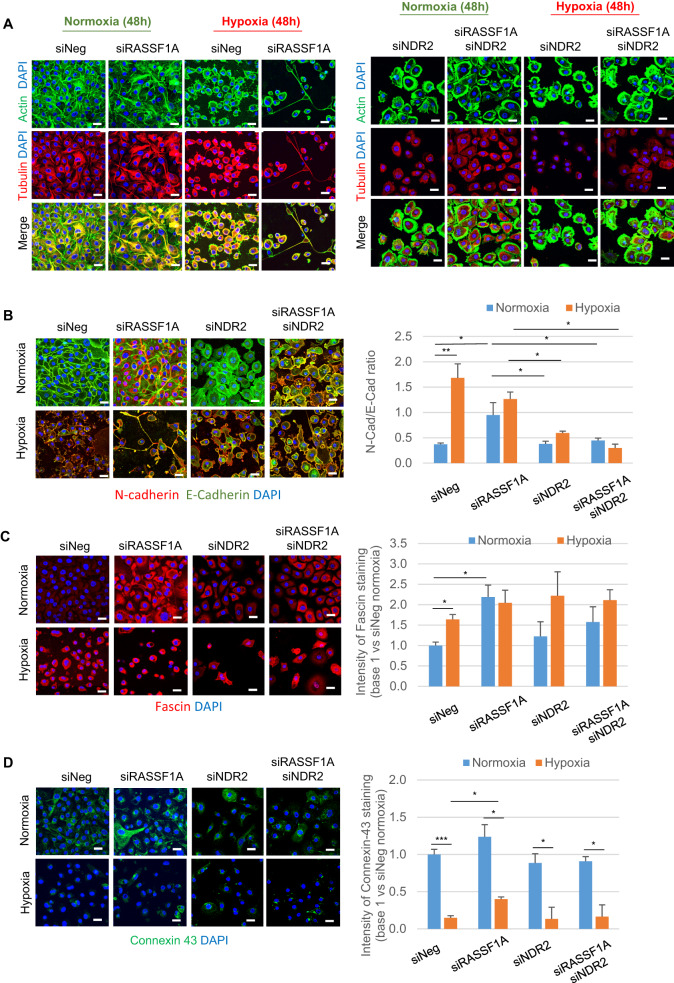
Severe and prolonged (0.2% O_2_, 48 h) hypoxia leads to epithelial-mesenchymal transition and cell junctions disrupt of HBEC-3 cells. **A**–**D** Immunostaining on HBEC-3 expressing or not RASSF1A and/or NDR2 after prolonged culturing (48 h) in normoxia/hypoxia showing (**A**) elements of the cytoskeleton, actin (green) and tubulin (red), (**B**) the N-cadherin (in red) and E-cadherin (green), (**C**) fascin (red), and (**D**) connexin 43 (Cx43) (green) (bare Scale 40 µm; *n* = 3). *P*-value *p < 0.05, **p < 0.01 (SEM *n* ≥ 3).

HBEC-3 cells grown in hypoxia are individualized and adopted stretched and/or even branched positions (Fig. 3A). We evaluate the effect of this hypoxia on the epithelial-mesenchymal transition, the adherent and communicating junctions and the elasticity of HBEC-3 cells by measuring the expression of E- and N-Cadherins [23], connexin-43 [24], and fascin [25] (Fig. 3B–D). E-cadherin (epithelial marker) decreases while *N*-Cadherin (mesenchymal marker) increases in HBEC-3 cells placed in hypoxia (0.2% O_2_, 48 h) compared to cells cultured in normoxia but not in RASSF1A- and/or NDR2-depleted HBEC-3 cells (Fig. 3B, RASSF1A and/or NDR2 depletion are confirmed by RASSF1A (Fig. S8A) and/or (Fig. S8B) NDR2 expression assay). In line, in hypoxia (0.2% O_2_, 48 h), fascin increases in HBEC-3 cells but not in RASSF1A- and/or NDR2-depleted HBEC-3 cells (Fig. 3C). Finally, connexin-43 decreases in hypoxia (0.2% O_2_, 48 h) in HBEC-3 cells with or without RASSF1A and/or NDR2 expression (Fig. 3D).

**Fig. 4:**
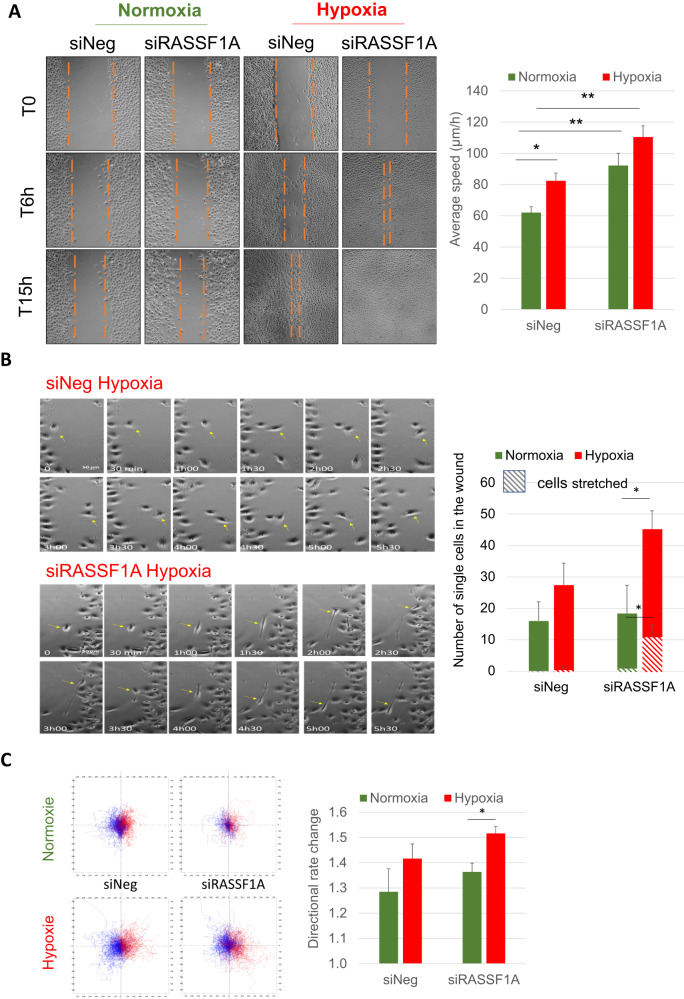
Severe and prolonged (0.2% O_2_, 48 h) hypoxia leads to elasticity increase of HBEC-3 cells and thus to increase in individual type migration. **A**, **B** Illustrations of the wound healing assay with HBEC-3 expressing or not RASSF1A, taken by inverted phase-contrast microscope (×10 magnification) in normoxia/hypoxia, at T0, T6 and T15h after scraping (**A**), zooming in on cell migration for conditions siNeg-hypoxia (**B**, upper panel) and siRASSF1A-hyopoxia (**B**, lower panel). **A** The average cell velocity (in µm/h) was measured in normoxia and hypoxia for the control conditions, in the absence of RASSF1A. *P*-value *p < 0.05, **p < 0.01 (SEM *n* ≥ 3). **C** Diagram representing the migration and the change of direction of HBEC-3, with in red the cells of the right edge of the wound and in blue those of the left edge (>300 cells) using the MtrackJ® module of the Fiji® software. Histograms represent directional rate change (i.e. change of angle between two coordinates (x, y) of the same cell at two different times) evaluated by TrackMATE® module of the Fiji® software for near 1000 cells per movie (SEM *n* ≥ 3). *P*-value *p < 0.05, **p < 0.01.

Control HBEC-3 cells (siNeg), grown in hypoxia (0.2% O_2_, 48 h), adopt an individual migration mode while their migration is collective in normoxia (Fig. 4A). This migration is of amoeboid type for the “control” cells (Fig. 4B) while mesenchymal for RASSF1A-depleted HBEC-3 cells (Fig. 4B). Since the type of migration (individual *versus* collective) influences the cell velocity [26], we measured the average velocity using the TrackMate module of the Fiji® software. These analyzes demonstrate that hypoxia (0.2% O_2_, 48 h), like the inactivation of RASSF1A, significantly increases the migration speed of HBEC-3 cells without additive effect (Fig. 4A). We evaluate the route of these cells using the MtrackJ® module of the Fiji® software, and show that compared to HBEC-3 cells in normoxia, HBEC-3 cells in hypoxia move randomly above all when depleted for RASSF1A (Fig. 4C).

**Fig. 5:**
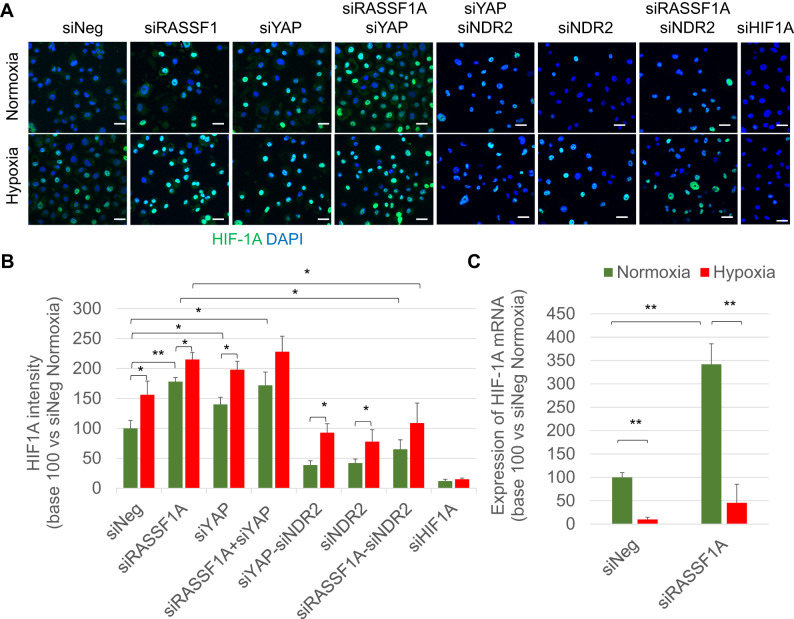
HIF-1A is activated by loss of RASSF1A and/or YAP expression in HBEC-3 cells cultured in hypoxia (0.2% O_2_, 48 h). Illustrations (**A**) and quantification (**B**) of HIF-1A on HBEC-3 expressing or not RASSF1A, YAP or HIF-1A (40 μm scale). **C** Expression of HIF1A mRNA measured by qPCR in HBEC-3 after 48 h of normoxia/hypoxia. Values are means ± SEM expressed in base 100 over siNeg in Normoxia. *P*-value *p < 0.05, **p < 0.01, and ***p < 0.001.

Hypoxia and RASSF1A depletion exhibit similar effect on increasing the velocity of HBEC-3 cells. Inactivation of YAP partly prevents the gain in migration velocity induced by RASSF1A depletion of HBEC-3 cells grown in hypoxia (0.2% O_2_, 48 h). As HIF-1A leads cell movements [27], *HIF-1A* expression was thus assessed in HBEC-3 at 48 h normoxia/hypoxia (protein level: Fig. 5A, B; mRNA level: Fig. 5C). RASSF1A depletion increases *HIF-1A* mRNA while this expression was significantly reduced in HBEC-3 cells grown in hypoxia (Fig. 5C).

Hypoxia and/or RASSF1A and/or YAP depletion activates the hypoxia factor HIF-1A. Indeed, the presence of nuclear HIF-1A in HBEC-3 cells at high cell density is reported whether cultured in normoxia or hypoxia (Fig. 5A). RASSF1A and/or YAP silencing enhances the nuclear intensity of HIF-1A but not in the absence of NDR2 (Fig. 5B). In line, hypoxia (0.2% O_2_, 48 h) increases the expression of *CAIX* (target gene of HIF-1A [28]) mRNA in HBEC-3 cells (Fig. S9) all the more so when cells are depleted for RASSF1A and/or YAP (Fig. S9).

The involvement of c-Jun in the positive effect of hypoxia on NDR2 and YAP was evaluated with an inhibitor of JNK, the SP600125. Indeed, c-Jun (1) is involved in cell motility, invasion and TEM [29–31], (2) cooperates with HIF-1 in hypoxia-induced gene transcription [32], (3) protects HIF-1A from degradation and is induced by prolonged or chronic hypoxia [33, 34], (4) is repressed by RASSF1A in lung cells [35], and (5) is a transcription factor for both YAP-1 (https://www.genecards.org/cgi-bin/carddisp.pl?gene=YAP1) and ARNT (https://www.genecards.org/cgi-bin/carddisp.pl?gene=ARNT).

**Fig. 6:**
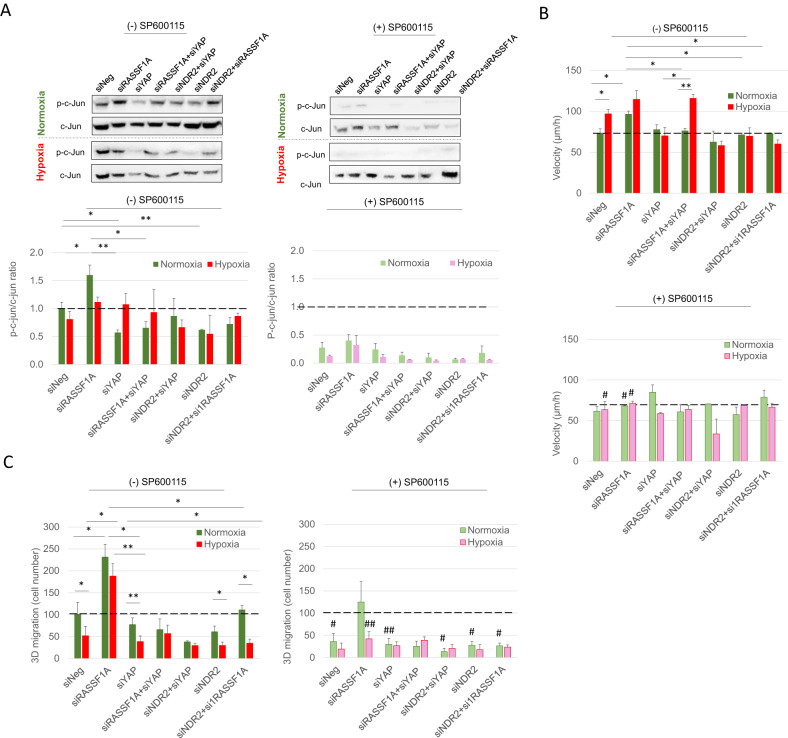
Hypoxia exacerbates the ability of HBEC cells with overactive NDR2 to migrate in a YAP and c-Jun-dependent mechanism. **A** Expression of c-Jun and phospho-c-Jun (p-c-Jun) proteins evaluated by western blot in HBEC-3 cells transfected with siNeg, siRASSF1A, siYAP, siNDR2, or both and cultivated for 48 h in normoxia or hypoxia (0.2% O_2_). Upper panels are representative experiments and lower panel is densitometric analysis of p-c-Jun/c-Jun ratio expressed in base 1 using siNeg in normoxia condition without SP600125. **B** Cells velocity and 3D migration (**C**) of HBEC-3 cells transfected with siNeg, siRASSF1A, siYAP, siNDR2 or both and cultivated for 48 h in normoxia or hypoxia (0.2% O_2_). The values are the mean ± SEM of three independent determinations. ANOVA was followed by a post-hoc Dunnett test, *p < 0.05, **p < 0.01 or #*p* < 0.05, ##*p* <using *t*-test by comparing (−) SP600125 vs (+) SP600125 in the same culture conditions.

In normoxia, the RASSF1A depletion increases phospho-c-Jun in HBEC-3 cells, while YAP and/or NDR2 silencing decreases phospho-c-Jun/c-Jun ratio and abrogates effect of RASSF1A depletion (Fig. 6A). In hypoxia, none of these effects are observed as, when cells were pre-treated with SP600125, a repressor of c-Jun activation. RASSF1A depletion significantly increases the HBEC-3 cells velocity (Fig. 6B) and 3D-migration (Fig. 6C) while hypoxia increases HBEC-3 cells velocity but decreases 3D-migration. YAP as NDR2 increases the velocity and migration induced by RASSF1A depletion in normoxia (Fig. 6B). In hypoxia, the increase of cell velocity and the decrease of 3D migration is still observed in the absence of RASSF1A and/or YAP expression but not in the absence of NDR2 (Fig. 6B). The inhibition of c-Jun activation abrogates or reduces the effects of RASSF1A depletion or hypoxia on HBEC-3 cell velocity (Fig. 6B) and 3D (Fig. 6C) migration respectively (NDR2 and/or RASSF1A depletion are confirmed by NDR2 (Fig. S10A) and/or RASSF1A (Fig. S10B) expression assay).

### NDR2 silencing strongly inhibits the xenograft formation and growth in a mice brain metastases model

**Fig. 7:**
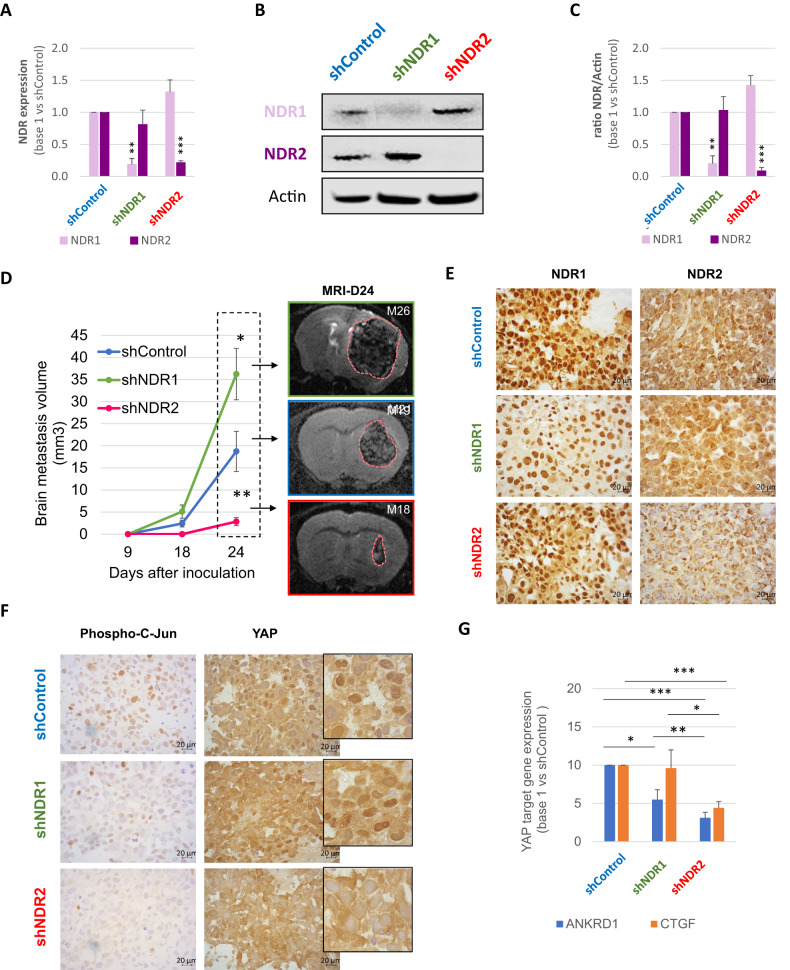
NDR2 silencing reduces development of brain metastases in Swiss Nude. **A**–**C** Silencing of NDR1 (shNDR1), NDR2 (shNDR2) or control (shControl) was confirmed by RTqPCR (**A**) and western blot (**B**, **C**) in H2030-BrM3. Mean ± SEM, *n* = 3, **p < 0.01 and ***p < 0.001 vs respective control. **D** Quantitative analyzes of tumor volume at 9, 18 and 24 days after cell inoculation in the left caudate putamen (striatum) from Swiss Nude (*n* = 10 animals per condition) using MRI. Mean ± SEM, *n* = 10 *p < 0.05 and **p < 0.01 vs Control group. Left panel show a representative T2w-MRI images of the lesions at D24 in the three different experimental groups. **E**–**G** Representative immunohistochemically analysis of NDR1 and NDR2 (**E**) or phospho-c-Jun and YAP (**F**) staining in brain metastases from the three experimental groups. **G** mRNA expression of YAP targets genes ANKRD1 and CTGF in H2030-BrM3 silenced for NDR1 (shNDR1), NDR2 (shNDR2) or control (shControl). Mean ± SEM, *n* = 3 *p < 0.05, **p < 0.01 and ***p < 0.001 vs respective control.

We use a lung cancer-derived brain metastases (BM) model in mice, and inoculated H2030-BrM3 cells (shControl, shNDR1 or shNDR2 (Fig. 7A–C)), in the right caudate putamen of Nude athymic mice (*n* = 10 per condition). At day 18, BM were observed in 6/10 and 7/10 animals in shControl and shNDR1 group respectively but not in shNDR2 group (Fig. 7D). At day 24, BM reached 36.22 ± 5.8 mm^3^ in shNDR1 group and 18.73 ± 4.5 mm^3^ in the shControl one (Table 1). In shNDR2 group, 6 animals started to developed BM at day 24, with significantly lower average volume (2.84 ± 0.9 mm^3^) compared to shControl and shNDR1 group (Representative image: Fig. 7D, all mice brain: Fig. S11). Immunostaining confirmed the lower expression of NDR1 and of NDR2 in BM of respective experimental groups (Fig. 7E). Comparable responses were obtained with the A549 xenograft (Table 1).

**Table 1:** NDR2 silencing decreases formation and growth rate of BM from HBEC.

	H2030-BrM3			A549	
	shControl	shNDR1	shNDR2	shControl	shNDR2
Animals with BM *n* (%)	7/10 (70%)	7/10 (70%)	6/10 (60%)	8/10 (80%)	5/9 (55.5%)
BM location					
Striatum	7/7 (100%)	7/7 (100%)	6/7 (85.7%)	8/8 (100%)	0
Cortex	0	2/7 (28.6%)	0	1/8 (12.5%)	5/5 (100%)
Start growing (day after inoculation)	19.71 ± 1.11	18.00 ± 0.00	24.00 ± 0.00^*^	33.0 ± 6.3	40.6 ± 5.5^*^
Growth rate (mm^3^/day)	18.73 ± 4.53	36.22 ± 5.81^*^	2.84 ± 0.90^**^, ^###^	7.27 ± 1.57	0.33 ± 0.13^**^

*t*-test : * vs shControl ; # vs shNDR1.

Furthermore, we observed a reduction of the nuclear staining of phospho-C-Jun and YAP in BM of shNDR2 group (Fig. 7F). In these cells, expression of YAP target genes (ANKRD1/CTGF) was reduced in comparison to the shControl or shNDR1 cell expression (Fig. 7G).

### NDR2 is more expressed in metastatic than in localized NSCLC

**Fig. 8:**
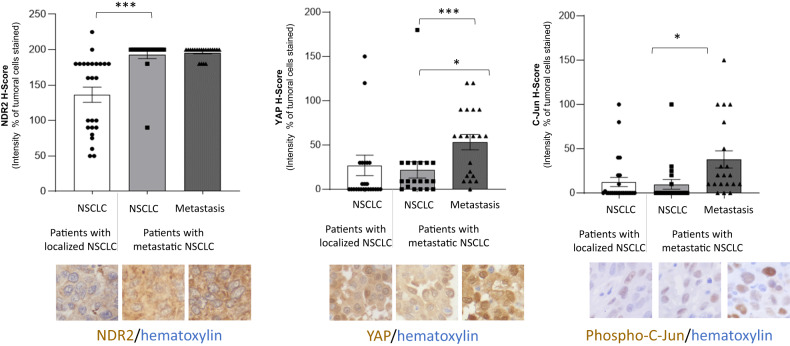
NDR2 expression increases in primitive NSCLC leading to brain metastasis. We immunostained a retrospective population of 45 patients operated on a non-metastatic NSCLC (*n* = 25) or metastatic NSCLC (*n* = 20) for whom both the primitive tumor and the brain metastasis (BM) were available, with NDR2 (1:400), YAP (1:400) or phospho-c-Jun (1:50). Data are the mean ± SEM of a IHC score calculated as the sum of the staining intensity (0–3) multiplied by the distribution (0–100%) (*p < 0.05, ***p < 0.001).

We assay NDR2, YAP and phospho-c-Jun expression in 25 patients with localized cancer and 20 patients with metastatic cancer (Fig. 8A–C). NDR2 is more expressed in tumor of metastatic NSCLC (H-score: 193.2 ± 5.8) than in localized NSCLC (136,4 ± 10,7). There was no difference in the expression of NDR2 between the primary and BM tumors of the same patients with NSCLC.

The nuclear YAP or phospho-c-Jun H-Score are similar between primary tumors of patients with localized NSCLC (YAP: 27.1 ± 11.6, phospho-c-Jun: 12.5 ± 5.3) and those with metastatic NSCLC (YAP: 21.9 ± 9.2, phospho-c-Jun: 9.8 ± 5.4). However, nuclear YAP or phospho-c-Jun are 2-fold higher in BM (YAP: 53.3 ± 8.6, phospho-c-Jun: 38.0 ± 9.7) than in primary tumor of patients with metastatic NSCLC.

## Discussion

We hypothesized that a hypoxic tumor microenvironment could contribute to the inactivation of the RASSF1A/Hippo pathway during bronchial tumor growth and underlies brain metastases formation. We first confirm that human primitive NSCLC as their brain metastases are hypoxic [20, 36–40]. Then, we report the ability of human bronchial epithelial cell (HBEC) lines expressing (HBEC-3, BEAS-2B) or not RASSF1A (A549, H1299, H1915, H2030-BRM3) to survive severe hypoxia at 0.2% oxygen which is consistent with the work having maintained cultures of HBEC in hypoxia (1% oxygen) for up to 28 days in an air-liquid interface [41].

We discovered that hypoxia inactivates TAZ in HBEC-3 cells but leads to the accumulation of active (dephosphorylated) nuclear YAP. Such results were recovered for the other HBEC lines with a few exceptions for BEAS-2B, H1299, and H1915 cell lines. For the BEAS-2B, this can be explained by the high basal level of YAP expression, probably due to their immortalization by SV40, an inhibitor of p53 which leads to the activation of YAP [42] which may not further increase. Under hypoxia, YAP is active in HBEC lines. That hypoxia act differently on YAP and TAZ was already described in ovarian cancer (5. The activation of YAP by hypoxia is supported by the silencing of its negative regulators: RASSF1A and Hippo kinases but not NDR2. That hypoxia inhibits Hippo kinases and promotes the nuclear localization of YAP as well as its transcriptional activity had already been reported in breast cancer [12], the liver [11], the colon [43], the pancreas [9], or the ovary [5] but not yet in lung cancer. Since hypoxia could influence the Hippo pathway through epigenetic modifications [8], we determined the methylation status of promoters of genes encoding members of the RASSF1A/Hippo pathway. We show that hypoxia does not induce methylation of the promoters from Hippo kinases or *ANKRD1* genes (ANKRD1 was studied since [22] reported frequent methylation of the promoter of this gene in bronchial tumors and that we observed a very strong transcription of this gene following hypoxia) nor does it demethylate the RASSF1A promoter in A549 cells. The decrease in expression of RASSF1A and of the kinases of the Hippo pathway induced by hypoxia does not therefore imply a modification of the methylation status of the promoters of the genes encoding these proteins. The mechanism of action remains to be determined but could involve ubiquitin-dependent regulations, as described in the breast cancer model in which SIAH2 directs the LATS2 kinase to the proteasome [7]. Indeed, all the members of the Hippo pathway are subject to regulation by ubiquitinylation [44].

YAP is described to transcribe genes involved in TEM and cell movement [15]. We evaluated the effect of hypoxia on the cytoarchitecture of HBEC-3 cells, their TEM and their 2D motility). The immunostaining of the actin and tubulin filaments confirmed that the RASSF1A depletion alters the cells morphology which become large or stretched in normoxia [15]. Here, we report in an original way that the alteration in cell morphology is enhanced when cells are grown under severe hypoxia. Again, that hypoxia alters the morphology of bronchial cells is in agreement with the work which reports that hypoxia affects the differentiation of HBEC in vitro: HBEC cells cultured at an air-liquid interface do not more succeed in forming cilia at their apex, and adopt a mucoid phenotype [41]. This change in morphology is in line with (i) the T (EM that we report in parallel in these cells, (ii) the fact that they undo their cell junctions (adherent and communicating), and (iii) the fact that they strongly express fascin, molecule known for its involvement in the formation of filopodia (fine cytoplasmic extensions) but also in the extensibility of cells [25] as well as cell migration [45]. This morphological/phenotypic change explains why HBEC adopt an individual type of migration when cultured in hypoxia and when they are brought to fill a mechanical wound made on their cell layer while in normoxia, their migration is collective. The hypoxia-induced amoeboid migration has only been described once to date and to our knowledge in a head and neck cancer model and is linked to HIF-1A [46]. We also observe that the HBEC grown in hypoxia do not efficiently repair the wound: HBEC move faster in hypoxia than in normoxia, but do not only migrate toward the other bank, particularly RASSF1A-depleted HBEC. This disorganized migration could be explained by the fact HBEC-3 cells grown in hypoxia and depleted for RASSF1A that strongly express the fascin which, as mentioned above, controls cell movement and elasticity. An increase in fascin has already been described in many cancers, in particular in NSCLC, where it predicts a poorer prognosis [47] because it promotes cell migration and invasion of NSCLC [48]. An increase in fascin has also already been described when cells are in hypoxia [49]. However, fascin is not a suitable therapeutic target since we observed that its inhibition by siRNA caused major cytonuclear abnormalities in HBEC (data not shown).

YAP is responsible for the increased collective migration rate induced by the RASSF1A depleted-HBEC cells cultured in normoxia [15]. We show here that, the migration velocity induced by the RASSF1A depletion is independent of YAP but could be dependent of NDR2 and HIF-1A which is stabilized by RASSF1A and/or YAP silencing in hypoxia. This result is unexpected since it was shown that RASSF1A stabilized HIF-1A in NSCLC cells [16] or that YAP stabilized HIF-1A [11]. It is therefore probable that the mechanisms allowing the stabilization of HIF-1A in the absence of RASSF1A or YAP are different, and could, for example, involve the transcription factor ETS-1 (v-ets erythroblastosis virus E26 oncogene homolog 1), which governs the gene expression of HIF-1A [50] and is itself activated by JNK signaling [51], which is repressed by RASSF1A [35]. Hypoxia experiments were carried out in percentage of O2 and for a duration different than that used by Dabral et al. [16], which suggests that the mechanisms are specific to a level of hypoxia (moderate, severe, chronic).

YAP activity in hypoxia coincides with the Hippo kinases decreases in hypoxia, except the NDR2 kinase, which appears to be stabilized, consistently with YAP nuclear localization in HBEC lines and YAP target genes expression. Indeed, NDR2 leads the YAP nuclear translocation in a GTPase RhoB pathway mechanism [14]. It would then be interesting to study the Rho protein pathway within the different lines to further understand how NDR2 promotes the YAP activation in hypoxia, hypoxia being also involved in cancer cell migration via the RhoA pathway [52].

We subsequently studied the expression of NDR2 and YAP on samples from patients with NSCLC. We show that NDR2 kinase expression is higher in metastatic than in localized NSCLC suggesting a link between the metastatic process and NDR2 expression. In addition, YAP is more frequently expressed higher in metastatic than in localized NSCLC. These observations highlight its potential role in the development of metastases, by inducing the expression of pro-metastatic target genes as has already been shown in breast cancer and melanoma cells [53]. The results also show that the expression of YAP at the nuclear level is higher in higher in metastatic than in localized NSCLC, which again suggests that the metastases take place in a YAP-dependent manner although this remains to be proven in an in-vivo study by carrying out an extinction of the expression of YAP or else by preventing its nuclear translocation.

Regarding the expression of YAP, the results did not reveal a significant difference in expression between the metastatic or non-metastatic NSCLC. Such comparison was not previously done by others to our knowledge but does not call into question the role of YAP in the formation of carcinoma metastases, in particular from lung, since independently of the quantity of YAP, the important thing is its activation.

We finally used a model of lung cancer-derived BM and show that silencing NDR2 kinase (but not NDR1) reduces the number of metastases and the overall volume and rate of lesion progression. Collectively, these results are therefore in favor of an effect of metastatic promotion of NDR2 and consistent with the role of the kinase NDR2 involved in the control of cell movement via the regulation of YAP demonstrating a pro-metastatic effect of the latter [15].

**Fig. 9:**
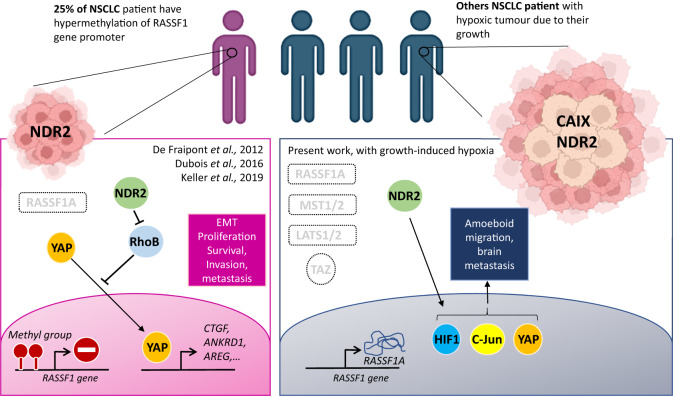
NDR2 underlies brain metastases from Non-Small Cell Lung Cancer. Hypoxia-induced activation of NDR2 underlies brain metastases from Non-Small Cell Lung Cancer (Graphical abstract).

In conclusion, our results demonstrate that hypoxia is an aggravating factor in bronchial carcinogenesis by silencing the RASSF1A/Hippo pathway (except NDR2) in HBEC lines. These new data improve our understanding of the relationship between the tumor microenvironment, the Hippo signaling pathway, and the adaptation of bronchial tumor cells. Our results shed light on HIF1 as a potential therapeutic target in patients with NSCLC with inactivation of the RASSF1A gene, but also YAP. Thus, the pharmacological targeting of these new targets could be effective in preventing the spread of cancer and in improving the vital prognosis of patients with NSCLC. Our results also indicated that NDR2 kinase is over-active in NSCLC in part by hypoxia and supports BM formation (Fig. 9). NDR2 expression is thus a useful biomarker to predict the metastases risk in patients with NSCLC, easily measurable routinely by immunohistochemistry on tumor specimens.

## Materials and methods

### Patients

We selected a retrospective population of 45 patients operated on a non-metastatic NSCLC (*n* = 25) or metastatic NSCLC (*n* = 20) for whom both the primitive tumor and the brain metastasis (BM) were available, at Caen University Hospital between December 2009 and December 2019. Among the 25 patients with localized NSCLC, 17 were men and 8 were women with an average age of 71 years [54 – 86 years]. Among the 20 patients with metastatic NSCLC, 15 were men and 5 were women with an average age of 67 years [40–82 years]. As required by French laws, all patients provided informed consent, and the study was approved by the institutional ethics committee (North-West-Committee-for-Persons-Protection-III N°DC-2008-588).

### Mice brain metastasis model

All animal investigations were performed under the current European directive (2010/63/EU) following ARRIVE guidelines, in the housing and laboratories #F14118001/#G14118001 and with the permission of the regional committee on animal ethics (C2EA-54 CENOMEXA, project #23280). Nude athymic mice (20-25 g, 8 weeks, male) were maintained in specific pathogen-free housing. Mice were manipulated under general anesthesia (5% isoflurane for induction, 2% for maintenance in a 1 l/min of 70%N_2_O/30%O_2_). Body temperature was monitored and maintained at 37.5 ± 0.5 °C throughout the experiments. Mice were placed in a stereotaxic head holder and a scalp incision was performed along the sagittal suture. H2030-BrM3 cells (10^5^ cells in 3-μl-PBS supplemented by glutamine (2 mM)) were injected over 5 min (0.6 µl/min) via a fine needle (30 G) connected to a Hamilton syringe. The injection sites were the right caudate putamen at a depth of 4 mm and lateralization on the right of 2.5 mm. Mice were randomly selected, and injected with H2030-BrM3 cells (shControl, shNDR1 or shNDR2). Animals were followed twice a week by anatomical MRI over a 21 days’ period to follow BM development. The endpoints of the protocol to limit pain of animals are described in the project authorization number#23280 (C2EA-54 CENOMEXA). The number of animals (*n* = 10/lot) was calculated to be as low as possible while allowing robust measurements (the distribution is Gaussian and parametric tests are applicable).

### Acquisition of magnetic resonance imaging and sequence analysis

The development of the lesions was monitored twice a week using magnetic resonance imaging (MRI) on a 7 Tesla magnet (Pharmascan, Bruker, Ettlingen, Germany). All experiments were performed under isoflurane anesthesia: 5% and during induction and 2.5% during the procedure in a 1 L/min mixture N20 and 02 (70 and 30%). The mouse is placed in a cradle allowing the head to be held by ear and tooth bars. Breathing is monitored in real time using a pressure balloon under the abdomen.

Fast imaging, FLASH sequence (Fast Low Angle Shot); TR/TEeff: 100/4 msec; resolution 0.39 × 0.39 × 3 mm^3^, acquisition time = 12 s), was out to verify the positioning of the animal and allow acquisition adjustments. T2-weighted imaging by rapid spin echo or RARE8 (Rapid Acquisition Relaxation Enhanced 8) sequence was then acquired with the following parameters: TR/TEeff = 5000/65 ms, number of repetitions = 1, spatial resolution = 0.078 × 0.078, 26 slices 0.5 mm, acquisition time = 2 min.

Tumor delineation was performed using ImageJ software (NIH, Wayne Rasband, Maryland, USA) on all adjacent T2w slices and tumor volume was achieved by multiplication of the sum of contiguous tumor surface areas with the slice thickness.

To note, the acquisition of MRI and sequence analysis were performed without knowing the group the animals belonged to (shControl, shNDR1 or shNDR2).

### Cell culture transfection and treatment

Immortalized human bronchial epithelial HBEC-3 cells, provided by Dr. Michael White (UT Southwestern Medical Center, Dallas, TX, USA), were grown as previously described [15]. BEAS-2B, A549, H1299, H1915 (from the American Type Culture Collection), and H2030-BrM3 (KRAS^G12C^ mutated from MSKCC, Dr Joan Massagué) were grown in Dulbecco’s Modified Eagle Medium (DMEM) supplemented with 10% (vol/vol) heat-inactivated fetal bovine serum. Mediums were complemented by 100 U/mL penicillin, 100 μg/mL streptomycin, and 2mM l-glutamine (Gibco, Life Technologies, Grand Island, NY, USA), and cells incubated at 37 °C in a humidified atmosphere with 5% CO_2_. Cells were routinely tested for mycoplasma contamination using MycoAlert® Mycoplasma Detection Kits (Lonza, Colmar, France), and recently authenticated by STR profiling (Microsynth AG, Switzerland).

RNAi oligonucleotides (Eurogentec®) sequences are in Table S1. Non-silencing negative control was from Dharmacon (Thermo Scientific, Pittsburgh, PA, USA). Plasmids are described [15]. The transfection was performed using Lipofectamine RNAiMax (Invitrogen, Carlsbad, CA, USA) in accordance with the manufacturer’s instructions at 30% (siRNA) and 70% (plasmids) of cell confluence.

For 0.2% oxygen culture, the cells were kept in a hypoxia workstation (INVIVO2, Ruskinn, ABE, Guipry, France) with an atmosphere humidified with 0.2% O_2_, 95% nitrogen and 5% CO_2_ at 37 °C.

For the c-jun pharmacological inhibition, the cells were treated with SP600125 (1 μM) (Selleckchem, Houton, TX, USA).

### Preparation of RNA and RT-PCR

The extraction of total RNA from cells was carried out using the illustra RNAspin mini® column (GE Healthcare, Bio-Sciences, Pittsburgh, PA, USA), according to the manufacturer’s instructions. Total RNA (250 ng) was reverse-transcribed with random primers and 200 IU M-MLV reverse transcriptase (37 °C, 90 min), followed by dissociation (70 °C, 5 min) with Mastercycler Eppendorf®. The resulting cDNAs were diluted (1/10) and used as templates. Polymerase chain reaction (PCR) was performed in a Mx3005P QPCR system (Agilent Technology, Les Ulis, France) with 5 pmol of each primer set (Table S2) and iQTM SYBR Green Supermix (Bio-Rad, Hercules, CA, USA). S16 was used as an internal control. Positive standards and reaction mixtures lacking the reverse transcriptase were employed routinely as controls for each RNA sample. Relative quantification was calculated using the ΔΔCt method.

### Preparation of DNA and methylation-specific PCR assay

DNA samples were obtained from cells using the QIAamp DNA Tissue kit (Qiagen, Les Ulis, France). Genomic DNA bisulfite modification was performed using the Epitect kit (Qiagen, Les Ulis, France), according the manufacturer’s instructions and previously described [54]. PCR was conducted with specific primers for either the methylated or unmethylated alleles (Table S3) in standard conditions.

### Antibodies, immunofluorescence, immunohistochemistry, immunoblotting, and image analysis

The antibodies are in Table S4.

For immunofluorescence, cells were seeded at a density of 2 × 10^4^ per 24-well, then washed with phosphate-buffered saline (PBS) and fixed with 4% paraformaldehyde (20 min, 37 °C). The cells were permeabilized with frozen methanol for 10 min and blocked with 4% bovine serum albumin for 1 h and stained with primary antibodies at 4 °C overnight. After wash with PBS, cells were stained with Alexa-488- or Alexa-555-conjugated secondary antibodies (Molecular Probes, Invitrogen, Eugene, OR, USA) (1 h, room temperature (RT)) and with DAPI (4,6 diamidino-2-phenylindole) (SantaCruz Biotechnology, Dallas, TX, USA). Digital pictures were captured using a high-throughput confocal microscopy (FluoView FV1000, Olympus).

For immunohistochemistry, tumor paraffin-embedded blocks were processed [55] with primary antibody diluted at 1:200. An overall IHC composite score was calculated (staining intensity (0–3, 0: negative, 1: weak, 2: moderate, and 3: strong) multiplied by the distribution (0–100%) from all parts of the slide).

For immunoblotting*,* whole-cell protein extracts were prepared [15], and proteins were detected by immunoblotting with the primary antibody diluted to 1:1 000 in Tween (0.1%)-TBS buffer and horseradish peroxidase (HRP)-conjugated secondary antibody, then revealed by enhanced chemiluminescence using the ECL kit (Promega™, Charbonnières-les-Bains, France). Densitometry results of western blot were analyzed with Image J software.

### BrdU incorporation assay

BrdU incorporation assay kit (Millipore, Billerica, MA, USA) was used in accordance with the manufacturer’s instructions. Spectrophotometric detection was performed at 450 nm wavelength.

### Caspase-3/7 assay

Caspase-3/7 activation was assayed using the Caspase-Glo 3/7 Luminescence Assay (Promega Corp., Madison, WI, USA) according to the manufacturer’s instructions.

### Wound-healing assay

Transfected cells were grown in complete medium onto 24-well Collagen IV coated plates (BD Biocoat™, Heidelberg, Germany). They were pretreated with mitomycin C at non-cytotoxic concentrations (1 ug/ml) for 12 h before an artificial “wound” carefully created at 0 h, using a P-20 pipette tip. Photographs were taken (X10) at 0 h and 6 h. The average velocity of cell migration was measured by subtracting distances across the wound at 0 h and 6 h and expressed as µm/h.

### 3D migration assay

In all, 25 × 10^3^ cells in 250 μl serum-free medium were added to the top chambers of 24-well Transwell plates containing a cell culture inserted with 8 μm pore size (Greiner Bio-One, Courtaboeuf, France). The lower chamber was filled with 700 μl complete media. After 48 h of incubation, the non-migrating cells (on the top) were removed and the migrating cells (on filter’s lower surface) were stained using crystal violet then counted under an inverted microscope at x20 magnification.

### Statistical analysis

Data are means ± SEM of three independent experiments. The data were analyzed using a two-tailed Student’s t-test (single comparison) or one-way ANOVA followed by Dunnett’s (multiple comparison analysis, GraphPad Prism 4, a GraphPad Software program (San Diego, CA, USA), the variance was similar between the groups that are being statistically compared). Differences are significant at *p* < 0.05.

### Supplementary information

Supplemental Figures

SUPPLEMENTAL TABLES

Original Western Blot

## Data Availability

All data are stored at the GIP Cyceron and Université de Caen Normandie (Caen, France), and can be made available upon request.
