# Supplementary material for: Hypoxia-induced activation of NDR2 underlies brain metastases from Non-Small Cell Lung Cancer
Source: Cell Death Dis. 2023 Dec 13;14(12):823. doi: 10.1038/s41419-023-06345-3 (PMC10719310; doi:10.1038/s41419-023-06345-3)
Supplement: Supplementary file 1 — Supplemental Figures [file 41419_2023_6345_MOESM1_ESM.pptx]

## Slide 1
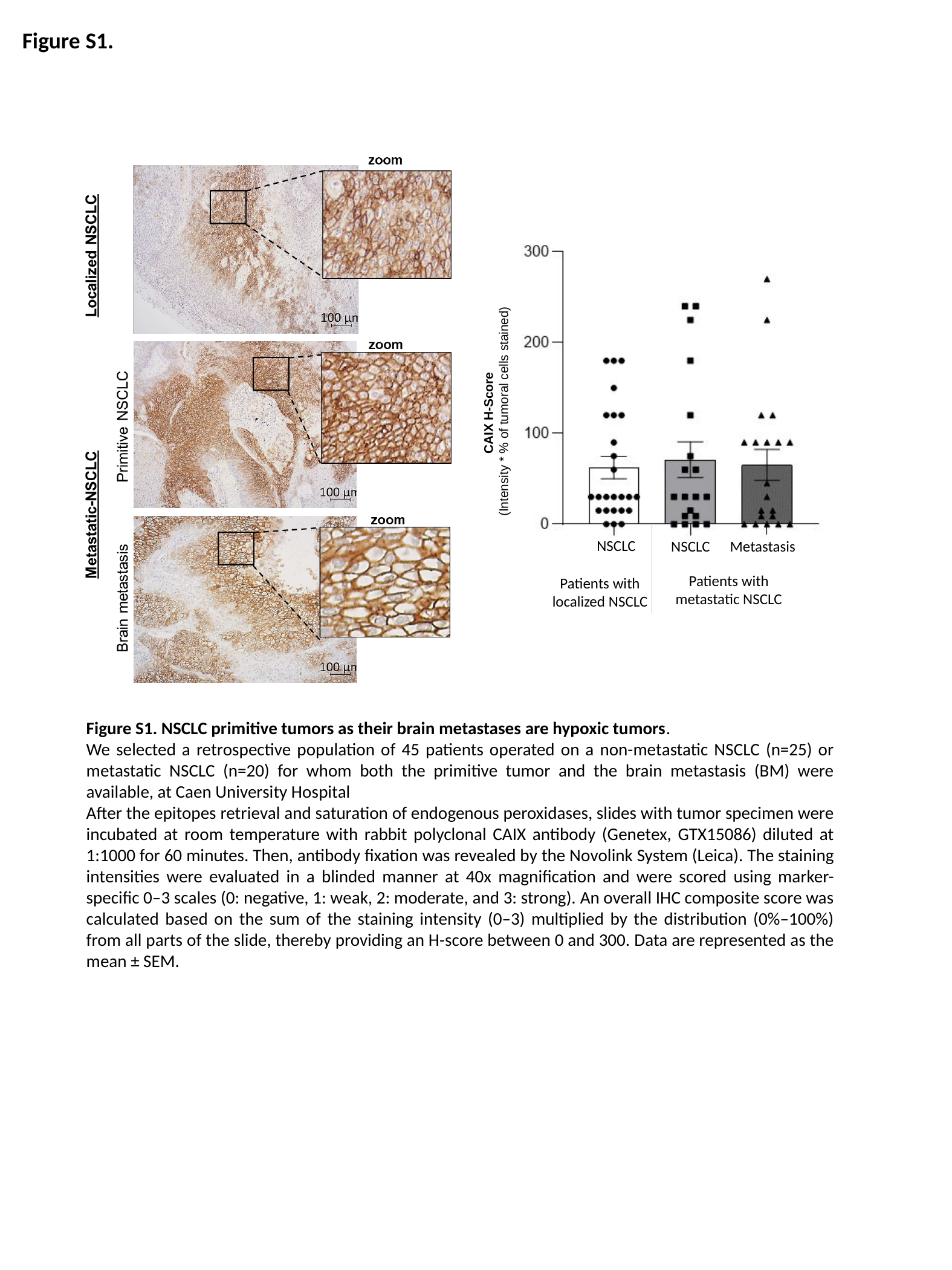

Figure S1.
CAIX H-Score
(Intensity * % of tumoral cells stained)
NSCLC
Metastasis
NSCLC
Patients with metastatic NSCLC
Patients with localized NSCLC
Figure S1. NSCLC primitive tumors as their brain metastases are hypoxic tumors.
We selected a retrospective population of 45 patients operated on a non-metastatic NSCLC (n=25) or metastatic NSCLC (n=20) for whom both the primitive tumor and the brain metastasis (BM) were available, at Caen University Hospital
After the epitopes retrieval and saturation of endogenous peroxidases, slides with tumor specimen were incubated at room temperature with rabbit polyclonal CAIX antibody (Genetex, GTX15086) diluted at 1:1000 for 60 minutes. Then, antibody fixation was revealed by the Novolink System (Leica). The staining intensities were evaluated in a blinded manner at 40x magnification and were scored using marker-specific 0–3 scales (0: negative, 1: weak, 2: moderate, and 3: strong). An overall IHC composite score was calculated based on the sum of the staining intensity (0–3) multiplied by the distribution (0%–100%) from all parts of the slide, thereby providing an H-score between 0 and 300. Data are represented as the mean ± SEM.

## Slide 2
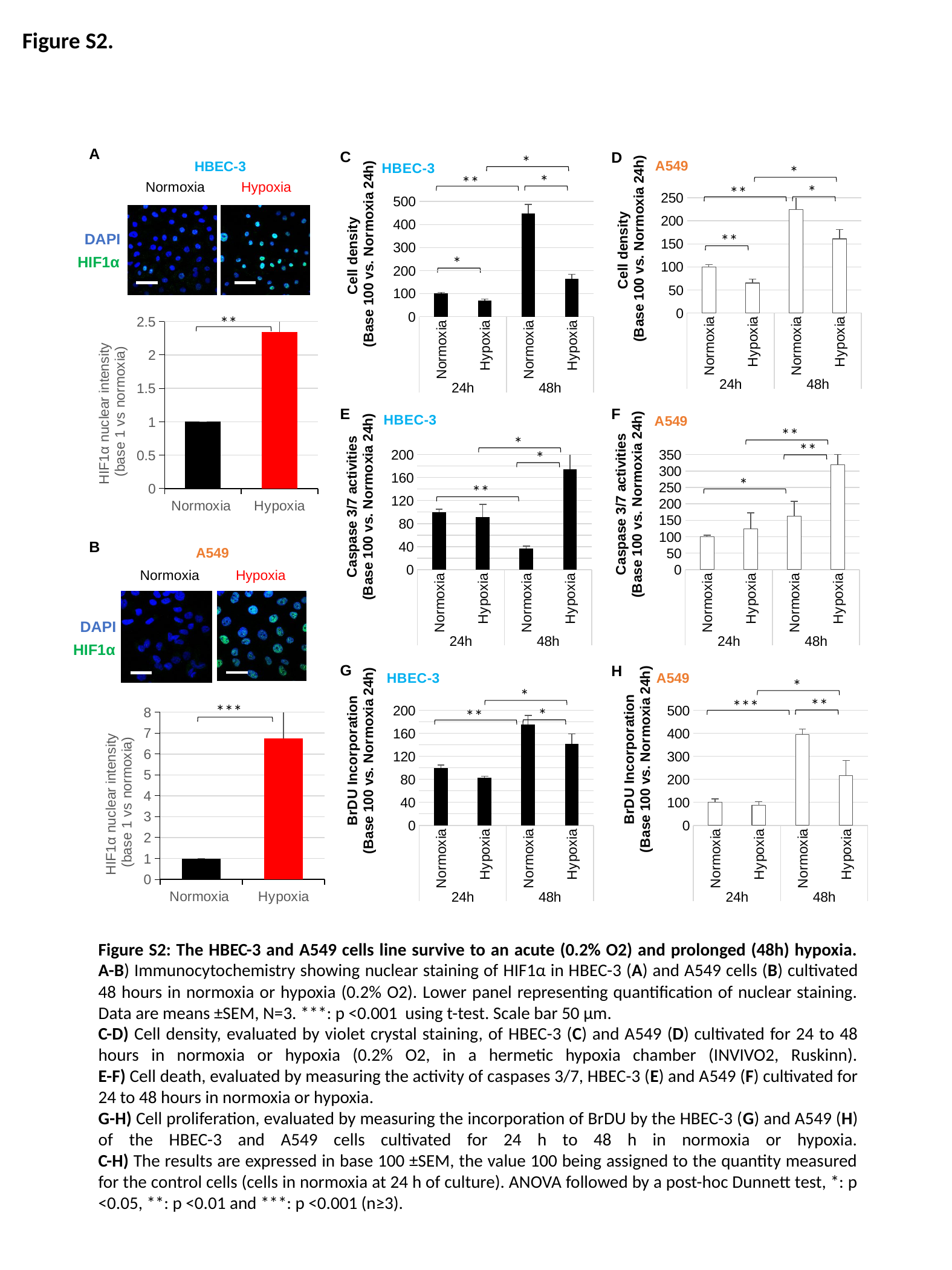

Figure S2.
A
C
D
*
### Chart: A549
| Category | pcDNA3 |
|---|---|
| Normoxia | 100.0 |
| Hypoxia | 64.9836152982124 |
| Normoxia | 225.05 |
| Hypoxia | 160.92 |HBEC-3
### Chart: HBEC-3
| Category | sineg |
|---|---|
| Normoxia | 100.0 |
| Hypoxia | 68.90283825945848 |
| Normoxia | 446.8570099391825 |
| Hypoxia | 164.80655898307506 |*
*
**
Normoxia
Hypoxia
*
**
DAPI
**
*
HIF1α
**
### Chart
| Category | HBEC |
|---|---|
| Normoxia | 1.0 |
| Hypoxia | 2.345450579283062 |E
F
### Chart: HBEC-3
| Category | sineg |
|---|---|
| Normoxia | 100.0 |
| Hypoxia | 91.73734112236218 |
| Normoxia | 36.166167990765075 |
| Hypoxia | 174.411689785046 |
### Chart: A549
| Category | pcDNA3 |
|---|---|
| Normoxia | 100.0 |
| Hypoxia | 123.82909020280698 |
| Normoxia | 162.74829420665614 |
| Hypoxia | 318.71189300988766 |
**
*
**
*
*
**
B
A549
Normoxia
Hypoxia
DAPI
HIF1α
G
H
### Chart: HBEC-3
| Category | sineg |
|---|---|
| Normoxia | 100.0 |
| Hypoxia | 82.58626126126128 |
| Normoxia | 174.74774160953407 |
| Hypoxia | 141.15595005382744 |
### Chart: A549
| Category | pcDNA3 |
|---|---|
| Normoxia | 100.0 |
| Hypoxia | 87.78417229384081 |
| Normoxia | 394.97067657633954 |
| Hypoxia | 215.64343447630736 |*
*
**
***
***
*
### Chart
| Category | A549 |
|---|---|
| Normoxia | 1.0 |
| Hypoxia | 6.732990993910376 |**
Figure S2: The HBEC-3 and A549 cells line survive to an acute (0.2% O2) and prolonged (48h) hypoxia.
A-B) Immunocytochemistry showing nuclear staining of HIF1α in HBEC-3 (A) and A549 cells (B) cultivated 48 hours in normoxia or hypoxia (0.2% O2). Lower panel representing quantification of nuclear staining. Data are means ±SEM, N=3. ***: p <0.001 using t-test. Scale bar 50 µm.
C-D) Cell density, evaluated by violet crystal staining, of HBEC-3 (C) and A549 (D) cultivated for 24 to 48 hours in normoxia or hypoxia (0.2% O2, in a hermetic hypoxia chamber (INVIVO2, Ruskinn).
E-F) Cell death, evaluated by measuring the activity of caspases 3/7, HBEC-3 (E) and A549 (F) cultivated for 24 to 48 hours in normoxia or hypoxia.
G-H) Cell proliferation, evaluated by measuring the incorporation of BrDU by the HBEC-3 (G) and A549 (H) of the HBEC-3 and A549 cells cultivated for 24 h to 48 h in normoxia or hypoxia.
C-H) The results are expressed in base 100 ±SEM, the value 100 being assigned to the quantity measured for the control cells (cells in normoxia at 24 h of culture). ANOVA followed by a post-hoc Dunnett test, *: p <0.05, **: p <0.01 and ***: p <0.001 (n≥3).

## Slide 3
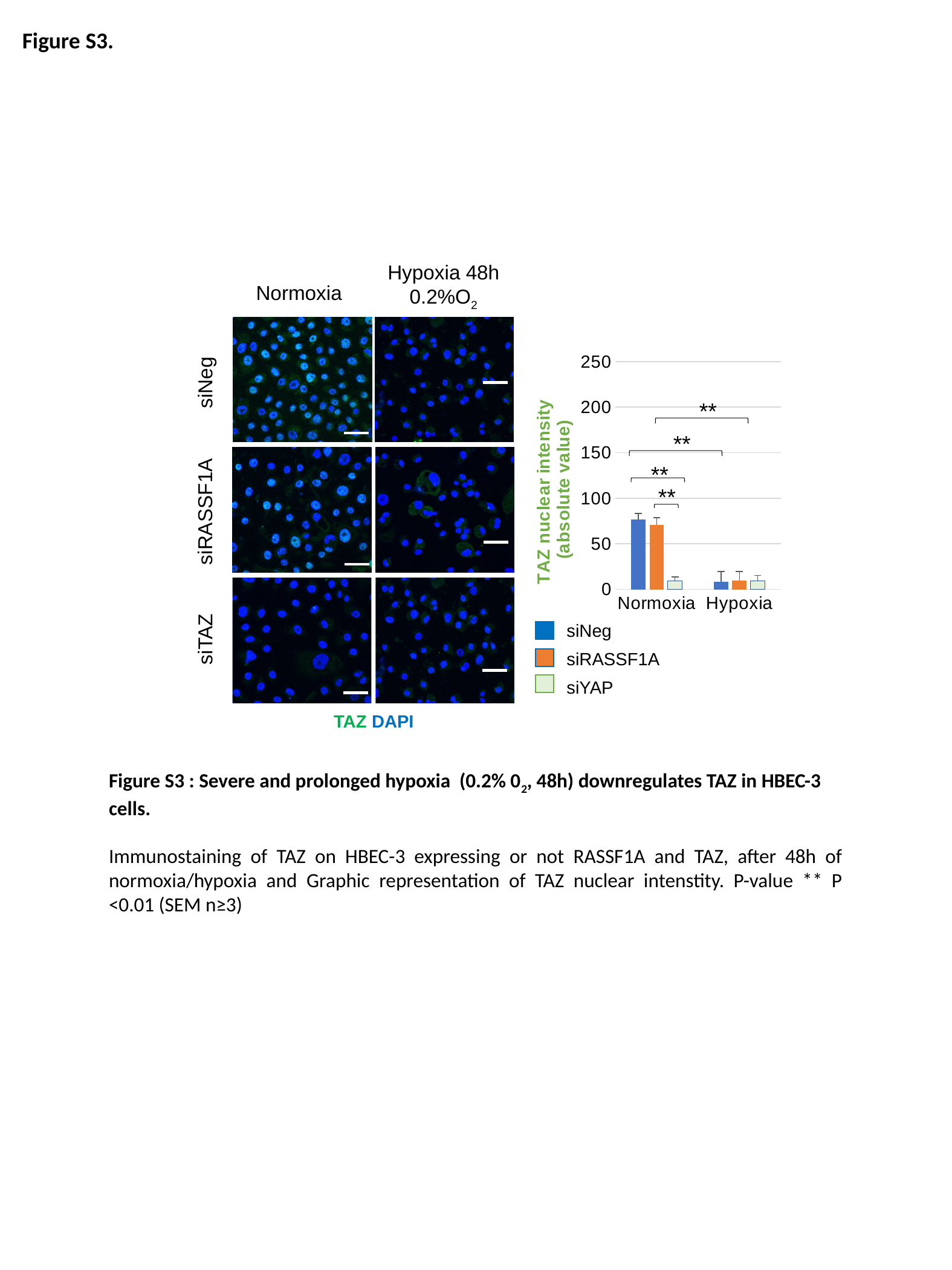

Figure S3.
Hypoxia 48h 0.2%O2
Normoxia
### Chart
| Category | siNeg | siRASSF1A | siTAZ |
|---|---|---|---|
| Normoxia | 76.51 | 70.62 | 9.5 |
| Hypoxia | 8.62 | 9.54 | 9.2 |siNeg
**
**
**
**
siRASSF1A
siNeg
siTAZ
siRASSF1A
siYAP
TAZ DAPI
Figure S3 : Severe and prolonged hypoxia (0.2% 02, 48h) downregulates TAZ in HBEC-3 cells.
Immunostaining of TAZ on HBEC-3 expressing or not RASSF1A and TAZ, after 48h of normoxia/hypoxia and Graphic representation of TAZ nuclear intenstity. P-value ** P <0.01 (SEM n≥3)

## Slide 4
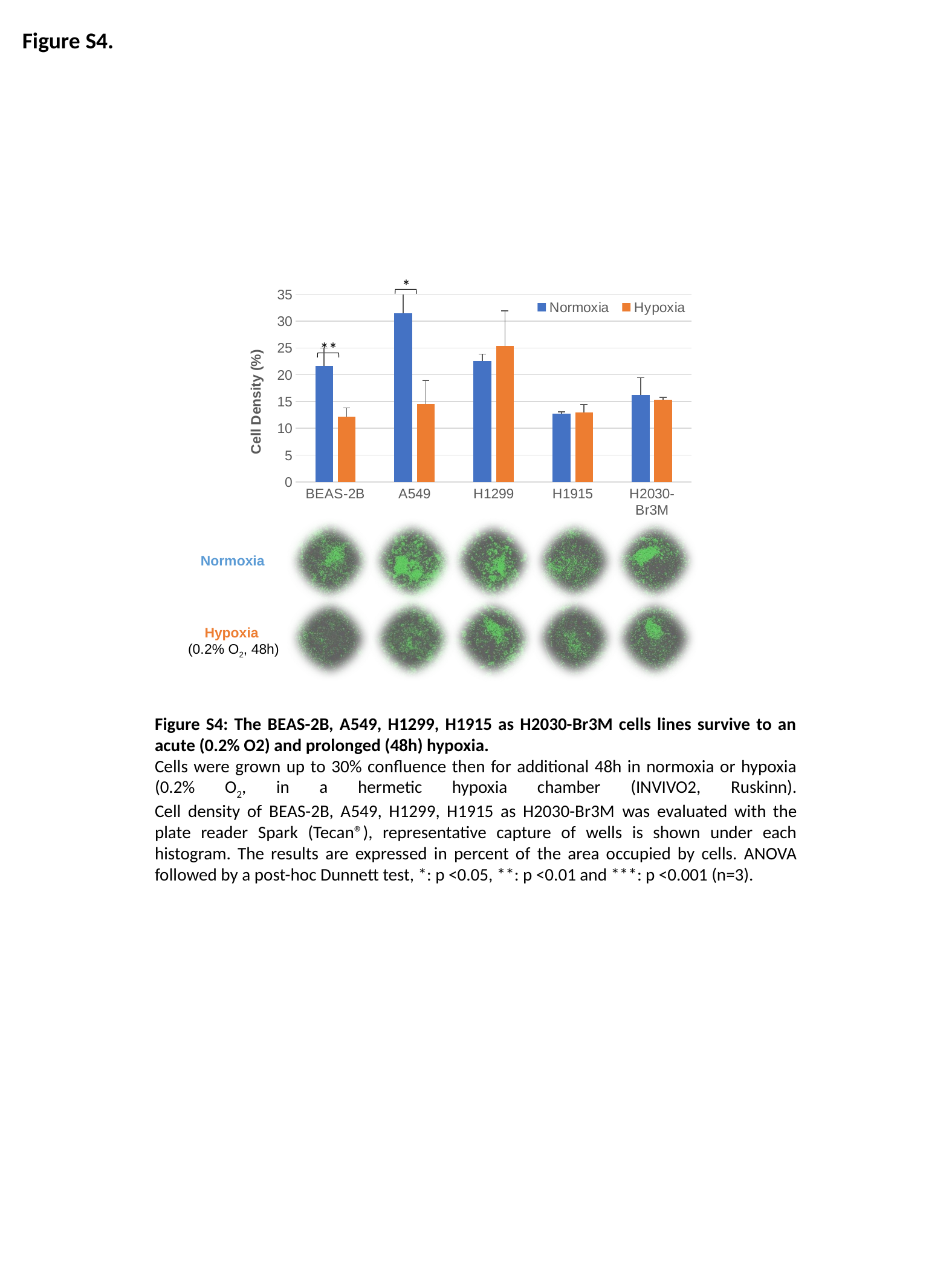

Figure S4.
*
### Chart
| Category | Normoxia | Hypoxia |
|---|---|---|
| BEAS-2B | 21.666666666666664 | 12.166666666666668 |
| A549 | 31.5 | 14.5 |
| H1299 | 22.583333333333336 | 25.333333333333336 |
| H1915 | 12.75 | 13.0 |
| H2030-Br3M | 16.25 | 15.333333333333332 |
**
Normoxia
Hypoxia
(0.2% O2, 48h)
Figure S4: The BEAS-2B, A549, H1299, H1915 as H2030-Br3M cells lines survive to an acute (0.2% O2) and prolonged (48h) hypoxia.
Cells were grown up to 30% confluence then for additional 48h in normoxia or hypoxia (0.2% O2, in a hermetic hypoxia chamber (INVIVO2, Ruskinn).
Cell density of BEAS-2B, A549, H1299, H1915 as H2030-Br3M was evaluated with the plate reader Spark (Tecan®), representative capture of wells is shown under each histogram. The results are expressed in percent of the area occupied by cells. ANOVA followed by a post-hoc Dunnett test, *: p <0.05, **: p <0.01 and ***: p <0.001 (n=3).

## Slide 5
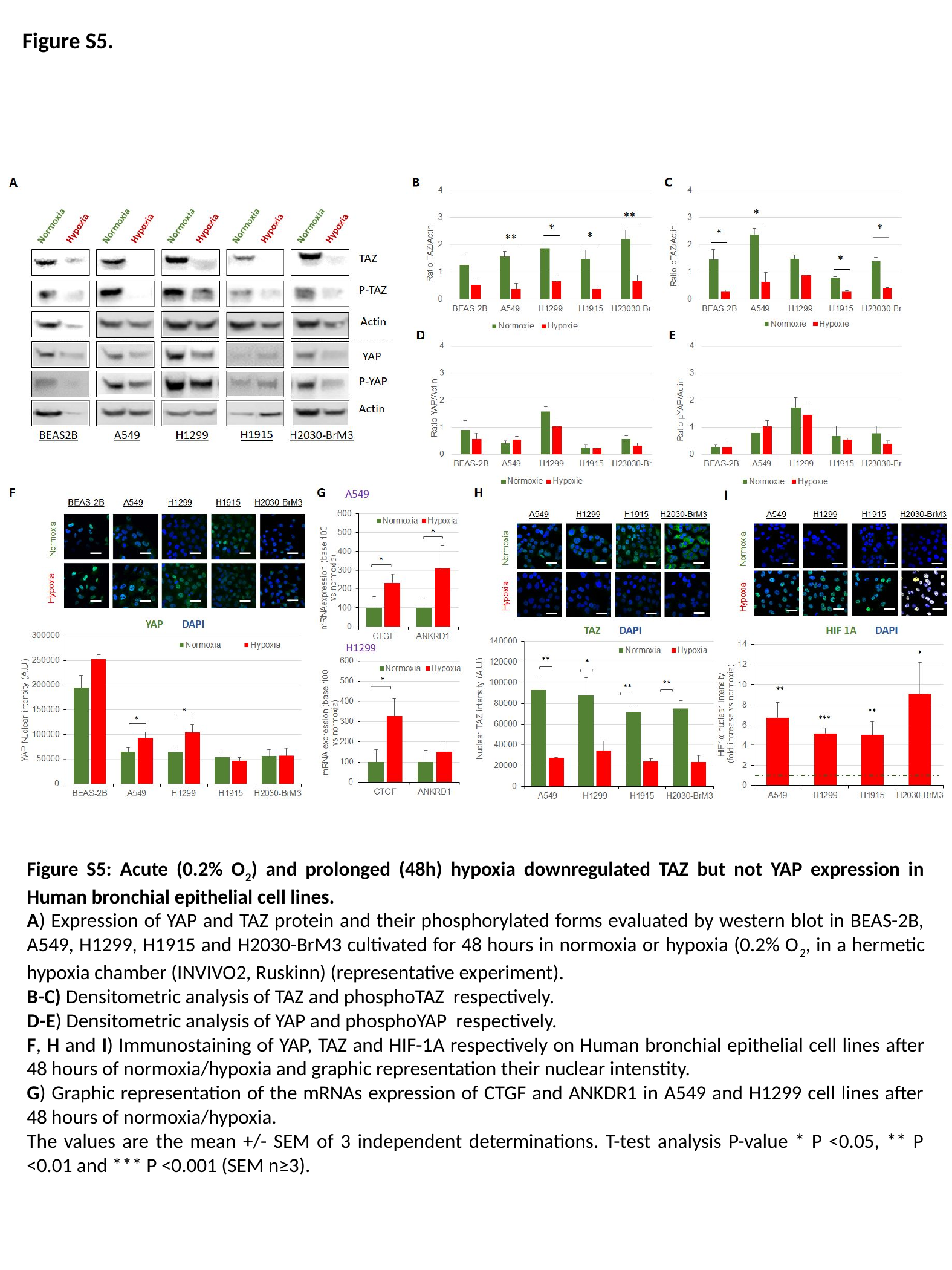

Figure S5.
Figure S5: Acute (0.2% O2) and prolonged (48h) hypoxia downregulated TAZ but not YAP expression in Human bronchial epithelial cell lines.
A) Expression of YAP and TAZ protein and their phosphorylated forms evaluated by western blot in BEAS-2B, A549, H1299, H1915 and H2030-BrM3 cultivated for 48 hours in normoxia or hypoxia (0.2% O2, in a hermetic hypoxia chamber (INVIVO2, Ruskinn) (representative experiment).
B-C) Densitometric analysis of TAZ and phosphoTAZ respectively.
D-E) Densitometric analysis of YAP and phosphoYAP respectively.
F, H and I) Immunostaining of YAP, TAZ and HIF-1A respectively on Human bronchial epithelial cell lines after 48 hours of normoxia/hypoxia and graphic representation their nuclear intenstity.
G) Graphic representation of the mRNAs expression of CTGF and ANKDR1 in A549 and H1299 cell lines after 48 hours of normoxia/hypoxia.
The values are the mean +/- SEM of 3 independent determinations. T-test analysis P-value * P <0.05, ** P <0.01 and *** P <0.001 (SEM n≥3).

## Slide 6
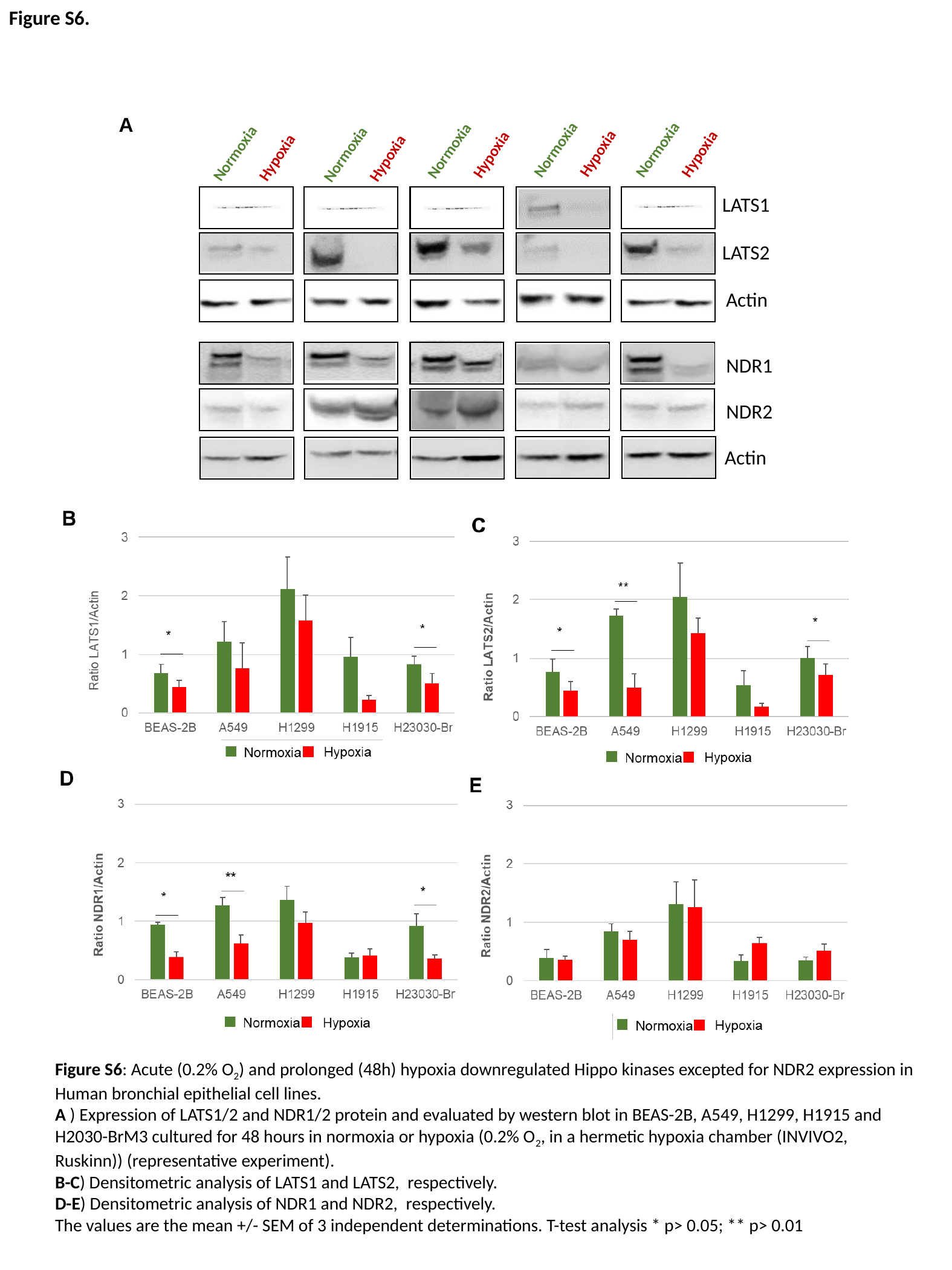

Figure S6.
A
Normoxia
Normoxia
Normoxia
Normoxia
Hypoxia
Hypoxia
Normoxia
Hypoxia
Hypoxia
Hypoxia
LATS1
LATS2
Actin
NDR1
NDR2
Actin
Figure S6: Acute (0.2% O2) and prolonged (48h) hypoxia downregulated Hippo kinases excepted for NDR2 expression in Human bronchial epithelial cell lines.
A ) Expression of LATS1/2 and NDR1/2 protein and evaluated by western blot in BEAS-2B, A549, H1299, H1915 and H2030-BrM3 cultured for 48 hours in normoxia or hypoxia (0.2% O2, in a hermetic hypoxia chamber (INVIVO2, Ruskinn)) (representative experiment).
B-C) Densitometric analysis of LATS1 and LATS2, respectively.
D-E) Densitometric analysis of NDR1 and NDR2, respectively.
The values are the mean +/- SEM of 3 independent determinations. T-test analysis * p> 0.05; ** p> 0.01

## Slide 7
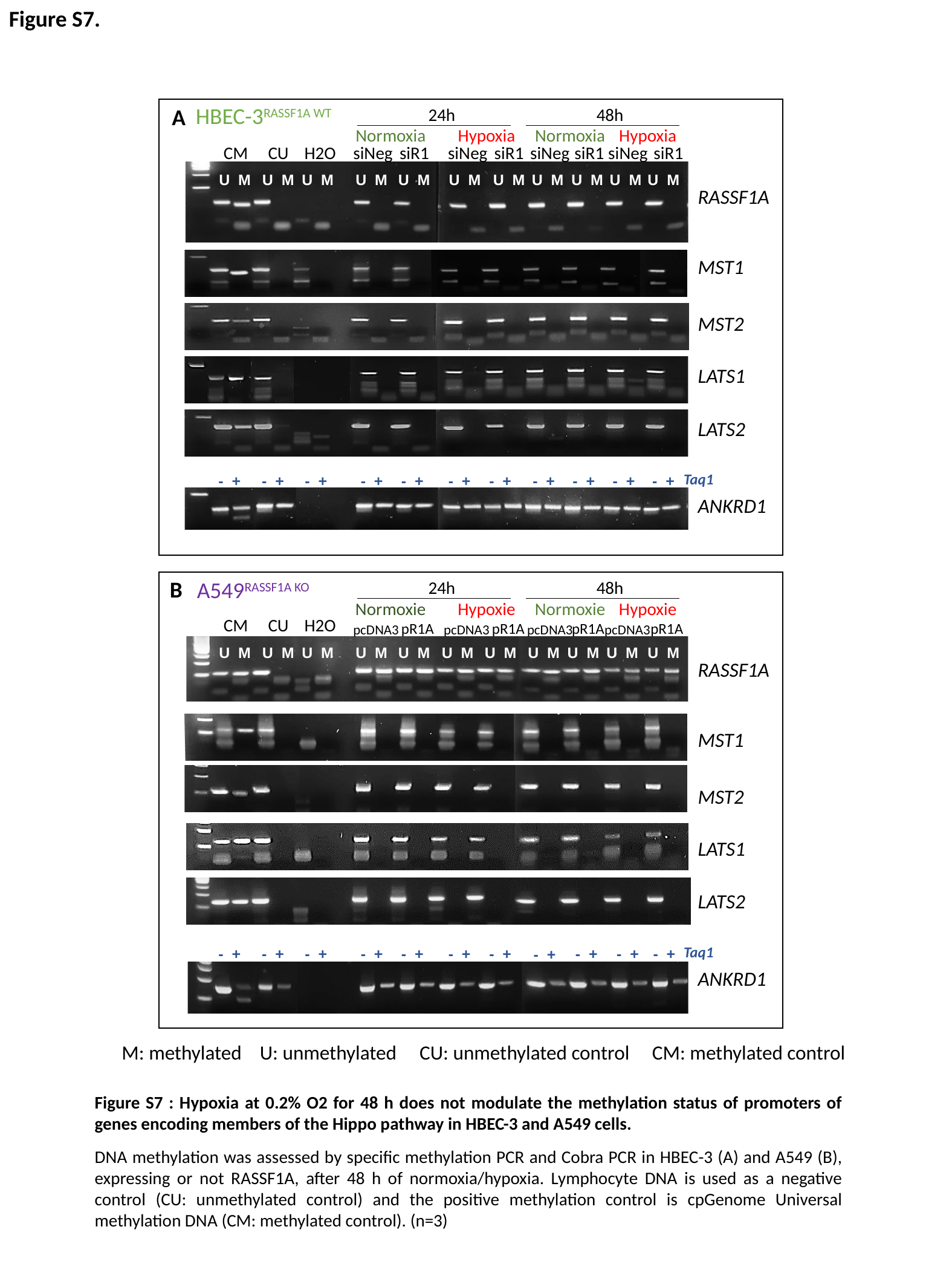

Figure S7.
HBEC-3RASSF1A WT
A
24h
48h
Normoxia
Hypoxia
Normoxia
Hypoxia
CM
CU
H2O
siNeg
siR1
siNeg
siR1
siNeg
siR1
siNeg
siR1
U M
U M
U M
U M
U M
U M
U M
U M
U M
U M
U M
RASSF1A
MST1
MST2
LATS1
LATS2
Taq1
- +
- +
- +
- +
- +
- +
- +
- +
- +
- +
- +
ANKRD1
B
A549RASSF1A KO
24h
48h
Normoxie
Hypoxie
Normoxie
Hypoxie
CM
CU
H2O
pR1A
pR1A
pR1A
pR1A
pcDNA3
pcDNA3
pcDNA3
pcDNA3
U M
U M
U M
U M
U M
U M
U M
U M
U M
U M
U M
RASSF1A
MST1
MST2
LATS1
LATS2
Taq1
- +
- +
- +
- +
- +
- +
- +
- +
- +
- +
- +
ANKRD1
M: methylated U: unmethylated CU: unmethylated control CM: methylated control
Figure S7 : Hypoxia at 0.2% O2 for 48 h does not modulate the methylation status of promoters of genes encoding members of the Hippo pathway in HBEC-3 and A549 cells.
DNA methylation was assessed by specific methylation PCR and Cobra PCR in HBEC-3 (A) and A549 (B), expressing or not RASSF1A, after 48 h of normoxia/hypoxia. Lymphocyte DNA is used as a negative control (CU: unmethylated control) and the positive methylation control is cpGenome Universal methylation DNA (CM: methylated control). (n=3)

## Slide 8
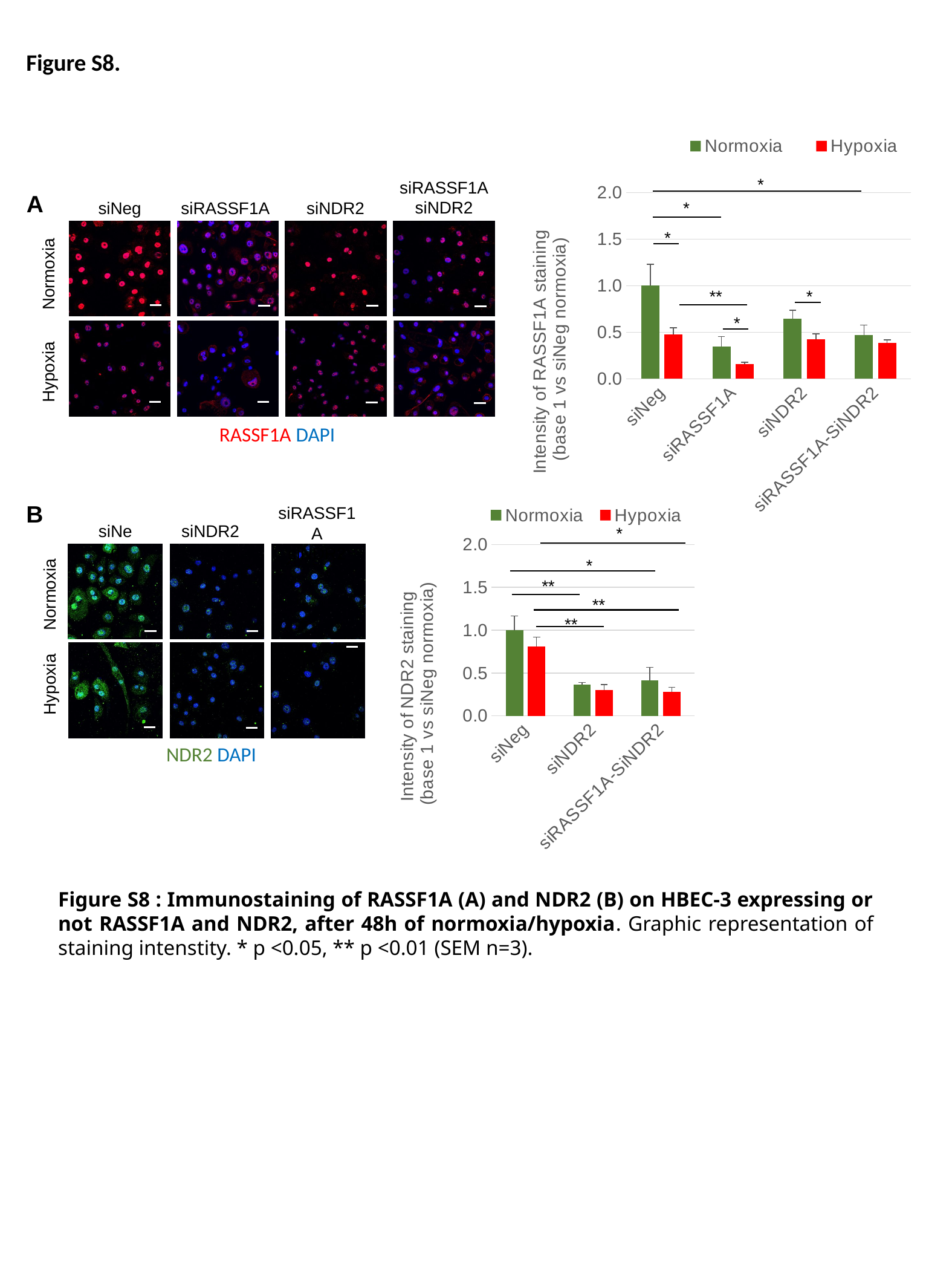

Figure S8.
### Chart
| Category | Normoxia | Hypoxia |
|---|---|---|
| siNeg | 10.0 | 4.740070193591764 |
| siRASSF1A | 3.4717749754886333 | 1.5800480870285716 |
| siNDR2 | 6.432963227565134 | 4.240558696037858 |
| siRASSF1A-SiNDR2 | 4.688260597331342 | 3.8360586433200345 |*
siRASSF1A
siNDR2
A
siRASSF1A
siNDR2
siNeg
*
*
Normoxia
**
*
*
Hypoxia
RASSF1A DAPI
B
siRASSF1A
siNDR2
### Chart
| Category | Normoxia | Hypoxia |
|---|---|---|
| siNeg | 10.0 | 8.120463097142707 |
| siNDR2 | 3.635808271682291 | 2.995411923072882 |
| siRASSF1A-SiNDR2 | 4.110789097546098 | 2.805667280983944 |siNeg
siNDR2
*
*
**
Normoxia
**
**
Hypoxia
NDR2 DAPI
Figure S8 : Immunostaining of RASSF1A (A) and NDR2 (B) on HBEC-3 expressing or not RASSF1A and NDR2, after 48h of normoxia/hypoxia. Graphic representation of staining intenstity. * p <0.05, ** p <0.01 (SEM n=3).

## Slide 9
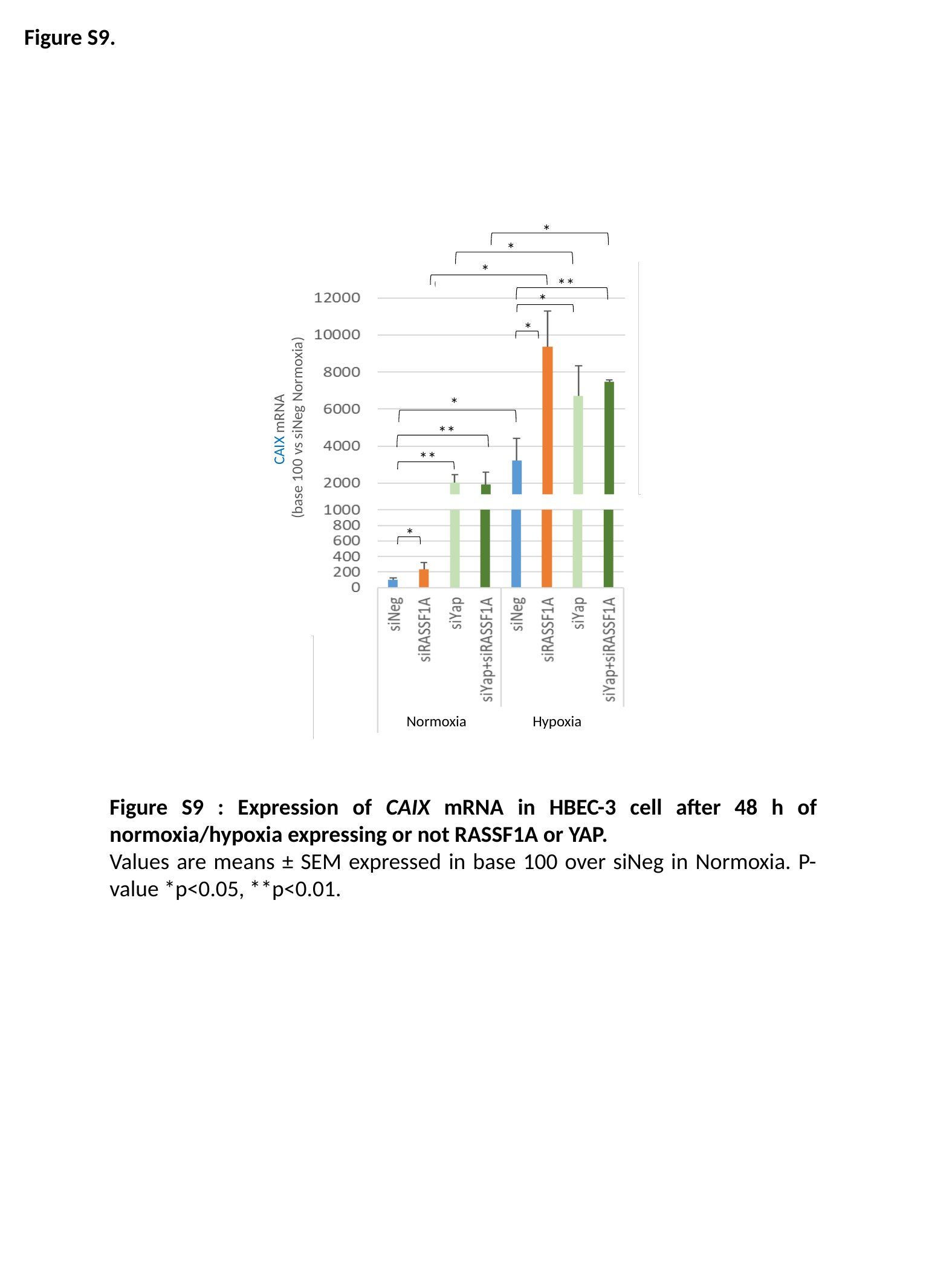

Figure S9.
*
*
*
**
*
*
*
CAIX mRNA
 (base 100 vs siNeg Normoxia)
**
**
*
Normoxia
Hypoxia
Figure S9 : Expression of CAIX mRNA in HBEC-3 cell after 48 h of normoxia/hypoxia expressing or not RASSF1A or YAP.
Values are means ± SEM expressed in base 100 over siNeg in Normoxia. P-value *p<0.05, **p<0.01.

## Slide 10
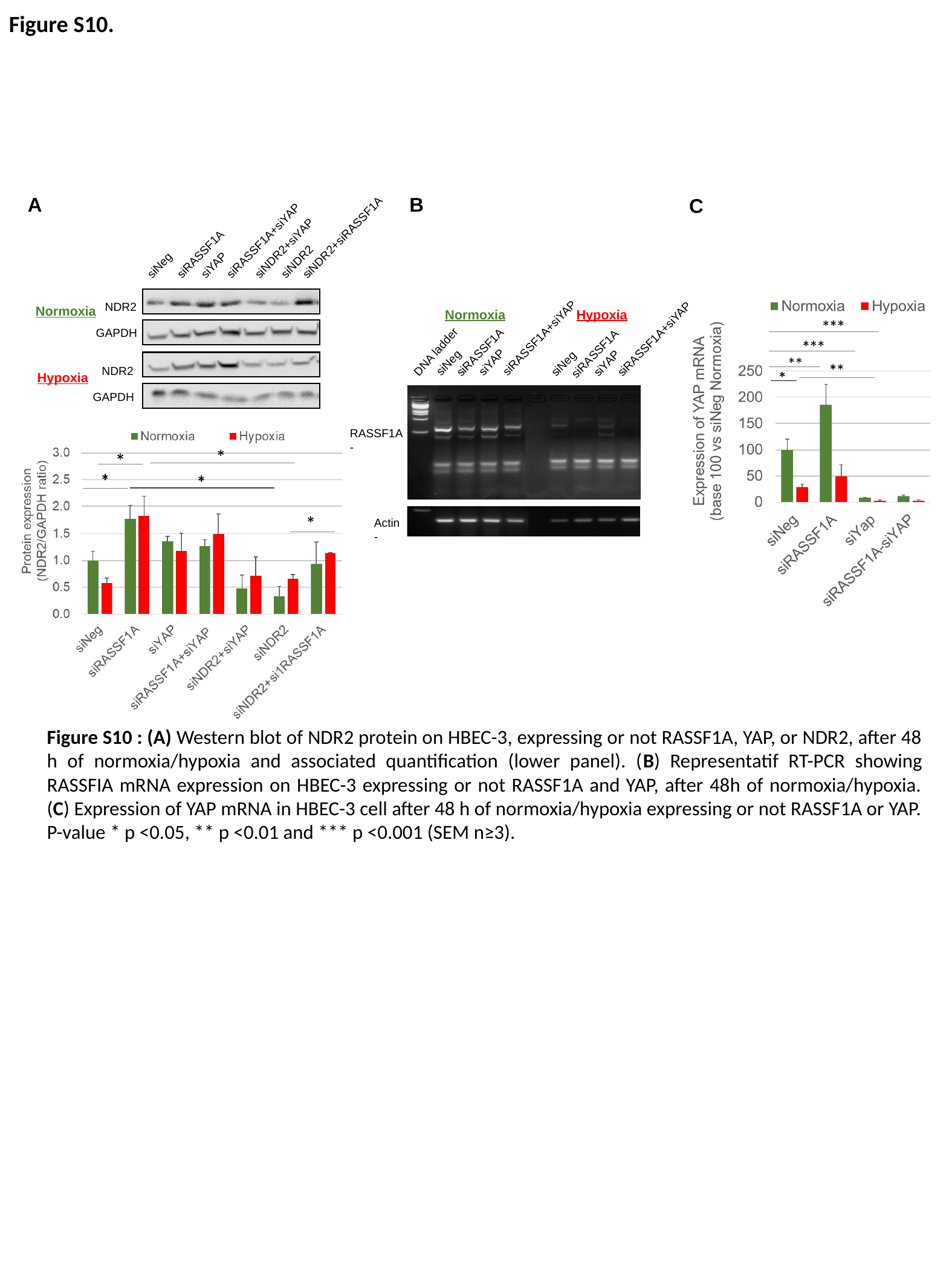

Figure S10.
A
B
C
siNDR2+siRASSF1A
siNDR2+siYAP
siRASSF1A+siYAP
siNDR2
siNeg
siRASSF1A
siYAP
NDR2
Normoxia
Normoxia
Hypoxia
GAPDH
siRASSF1A+siYAP
siRASSF1A+siYAP
siNeg
siYAP
siRASSF1A
siNeg
siYAP
DNA ladder
siRASSF1A
NDR2
Hypoxia
GAPDH
RASSF1A -
Actin -
Figure S10 : (A) Western blot of NDR2 protein on HBEC-3, expressing or not RASSF1A, YAP, or NDR2, after 48 h of normoxia/hypoxia and associated quantification (lower panel). (B) Representatif RT-PCR showing RASSFIA mRNA expression on HBEC-3 expressing or not RASSF1A and YAP, after 48h of normoxia/hypoxia. (C) Expression of YAP mRNA in HBEC-3 cell after 48 h of normoxia/hypoxia expressing or not RASSF1A or YAP. P-value * p <0.05, ** p <0.01 and *** p <0.001 (SEM n≥3).

## Slide 11
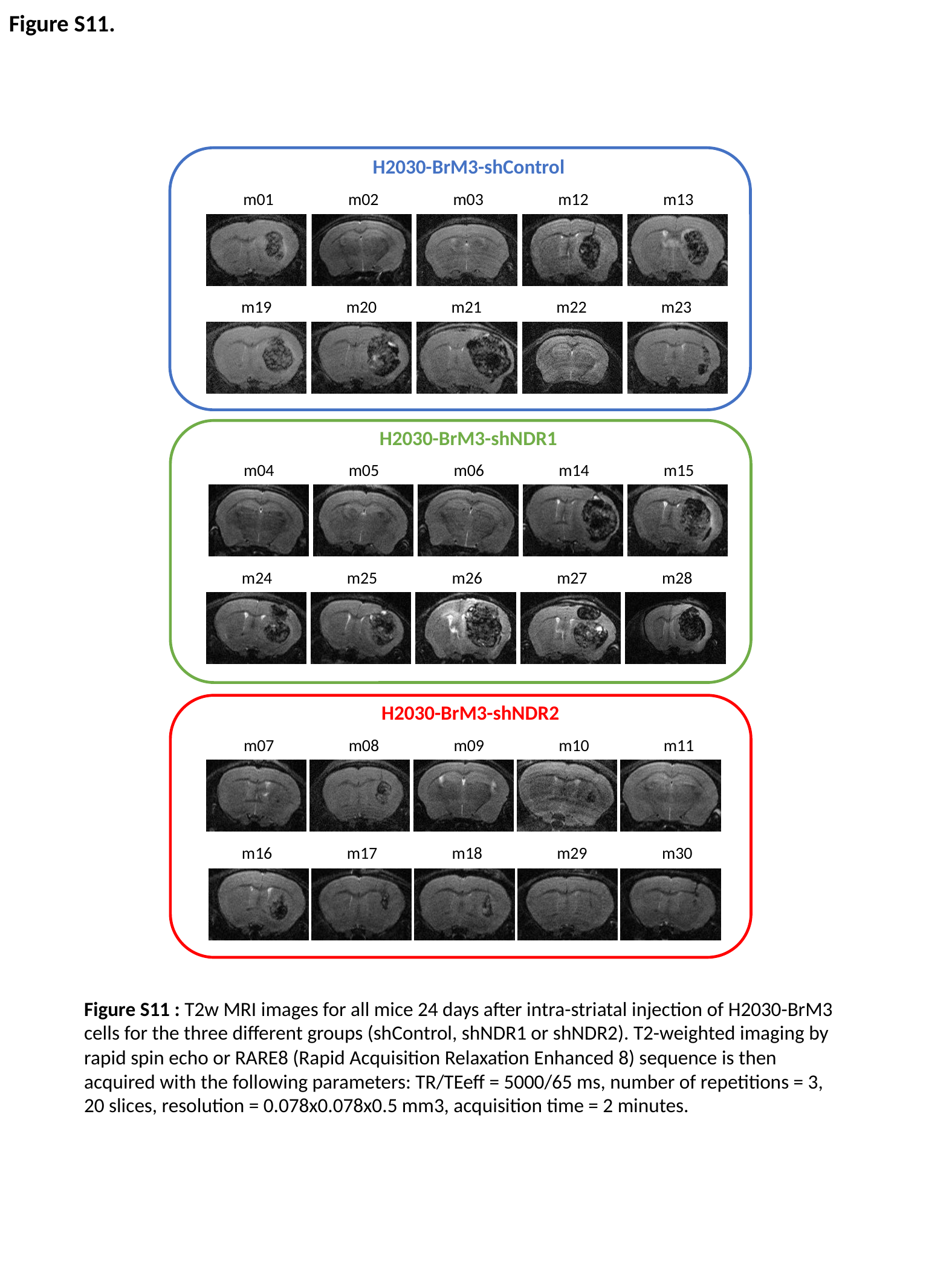

Figure S11.
H2030-BrM3-shControl
m01
m02
m03
m12
m13
m19
m20
m21
m22
m23
H2030-BrM3-shNDR1
m04
m05
m06
m14
m15
m24
m25
m26
m27
m28
H2030-BrM3-shNDR2
m07
m08
m09
m10
m11
m16
m17
m18
m29
m30
Figure S11 : T2w MRI images for all mice 24 days after intra-striatal injection of H2030-BrM3 cells for the three different groups (shControl, shNDR1 or shNDR2). T2-weighted imaging by rapid spin echo or RARE8 (Rapid Acquisition Relaxation Enhanced 8) sequence is then acquired with the following parameters: TR/TEeff = 5000/65 ms, number of repetitions = 3, 20 slices, resolution = 0.078x0.078x0.5 mm3, acquisition time = 2 minutes.
