# Supplementary material for: Hypoxia-induced activation of NDR2 underlies brain metastases from Non-Small Cell Lung Cancer
Source: Cell Death Dis. 2023 Dec 13;14(12):823. doi: 10.1038/s41419-023-06345-3 (PMC10719310; doi:10.1038/s41419-023-06345-3)
Supplement: Supplementary file 2 — SUPPLEMENTAL TABLES [file 41419_2023_6345_MOESM2_ESM.docx]

**Table.S1.** siRNA used in this work.

| Target | siRNA (5’ ⭢ 3 ‘) | ref |
| --- | --- | --- |
| RASSF1A | **si1:** GACCUCUGUGGCGACUUCA(TT)  **si2:** GAACGUGG ACGAGCCUGU(TT) | (15) |
| NDR1 | **si1:** AAGUAAUAGGCAGAGGAGCAU(TT)  **si2:** AAGAGCAGGUUGGCCACAUUC(TT) | (14) |
| NDR2 | **si1:** AAGUUACGUCGAUCACAACAC(TT)  **si2:** AAGACACCUUGACAGAAGAGG (TT) | (14) |
| YAP | **si1:** UGAGAACAAUGACGACCAA (TT)  **si2:** CCACCAAGCUAGAUAAAGA (TT) | (14) |
| TAZ | **si1:** AGGUACUUCCUCAAUCACA (TT)  **si2:** CUAGGAAGGCGAUGAAUCA (TT) | (14) |
| HIF1-a | **si1:** CACCUAUGACCUGCUUGGUGCUGAU (TT)  **si2:** CUGAUGACCAGCAACUUGA (TT) | [56-58] |
| HIF2-a | si1: CAGGUGGAGCUAACAGGACAUAGUA (TT)  si2: GGUUUUGUUGCUAGCCCUU (TT) | [56-58] |

**Table.S2.** Primers used for PCR and qRT-PCR in this work.

| PCR | Target | Primers (5’ ⭢ 3 ‘) |
| --- | --- | --- |
| PCR | RASSF1A | Forward (F): GGG GTC GTC CGC AAA GGC C  Reverse (R): GGG TGG CTT CTT GCT GGA GGG |
|  | LATS2 | F: TCT GTG ACT GGT GGA GTG TTG  R: CTT CTC CTG AAG GCT TTG |
|  | Actin | F: CAA CCG TGA AAA GAT GAC CCA G  R: ATG GGC ACAGTG TGG GTG AC |
| qRT-PCR | MST1 | F: GTA GCC AGC ACC ATG ACT GA  R: TTG CCA AAG CTG TTG ATC TG |
|  | MST2 | F: ctg agt gaa gac agt ttg act  R: CGA CAA CTT GAC CGG ATT CC |
|  | LATS1 | F: TGG TCA TAT TAA ATTGAC TGA C  R: CCA CAT CGA CAG CTT GAG GG |
|  | YAP | F: GCC GGA GCC CAA ATC C  R: GCA GAG AAG CTG GAG AGG AAT G |
|  | TAZ | F: ACC CAC CCA CGA TGA CCC CA  R: GCA CCC TAA CCC CAG GCC AC |
|  | CTGF | F: ACG AGT GGG TGT GTG ACG A  R: CCA GGC ACT TGG CTC TAA TC |
|  | AREG | F: TCC CGA GGA CGG TTC ACT AC  R: TCC CGA GGA CGG TTC ACT AC |
|  | ANKDR1 | F: AGT AGA GGA ACT GGT CAC TGG  R: TGG GCT AGA AGTGTC TTC |
|  | HIF1a | F: CCA GCA GAC TCA AAT ACA AGA ACC  R: TGT ATG TGG GTA GGA GAT GGA GAT |
|  | HIF2a | F: GCA TGG ACA TGA AGT TCA CCT AC  R: CCA CTT ACT ACC TGA CCC TTG |
|  | CAIX | F:TGG AGA GGA GGA TCT ACC  R: GCA TTA TTC TGG GGT TCT TGA G |
|  | S16 | F: CTG GAG CCA GTT CTG CTT CT  R: TCT GGT AAT AGG CCA CCA GG |

**Table.S3.** Primers used for MS-PCR in this work.

|  | **Sequence (5’⭢3’)** | **T_M_** | **Size (bp)** | **Reference** |
| --- | --- | --- | --- | --- |
| ***MST1/STK4* (Genbank access : NC_000020.11)** | | | | |
| **U:** | **F:** TTTGTGGGGTGGGTTTAGGAGGTTTGT | 63°C | 125 | [59] |
|  | **R:** AACCAATAACCCCTCACCAACACAACAA |  |  |  |
| **M:** | **F:** GCGGGGCGGGTTTAGGAGGTTC |  | 120 |  |
|  | **R:** CCAATAACCCCTCACCGACGC |  |  |  |
| ***MST1/STK3* (Genbank access : NC_000008.11)** | | | | |
| **U:** | **F:** TTTTAAGTGGGAGGGAGATTTGTTGTGG | 61°C | 108 | [59] |
|  | **R:** AAAAACCAAAACACCAACCAACCAAACC |  |  |  |
| **M:** | **F:** CGGGAGGGAGATTCGTCGCG | 63°C | 99 |  |
|  | **R:** AAACCGAAACACCGACCGACCG |  |  |  |
| ***LATS1* (Genbank access : NC_000006.12)** | | | | |
| **U:** | **F:** TGAATGATTAGAGTTGTGGGTGATGT | 60°C | 128 | [59] |
|  | **R:** AAACATTTCCCAACATCACTTACACA |  |  |  |
| **M:** | **F:** GAACGATTAGAGTTGCGGGCGAC | 62°C | 126 |  |
|  | **R:** AACATTTCCCGACGTCGCTTACG |  |  |  |
| ***LAST2* (Genbank access : NC_000013.11)** | | | | |
| **U:** | **F:** GGTGTTTTGTTTGGATTGGTATGTGGTT | 60 °C | 141 | [59] |
|  | **R:** CATCTTCCCAAAACACTCACACCACA |  |  |  |
| **M:** | **F:** TTCGTTCGGATTGGTATGCGGTC |  | 137 |  |
|  | **R:** CCATCTTCCCGAAACGCTCACG |  |  |  |
| ***NDR1/STK38* (Genbank access : NC_000006.12)** | | | | |
| **U:** | **F:** AATTAGTTTGGTTTTTATTAGGTGG | 59°C | 149 | MethPrimer |
|  | **R:** AATAACTAAATCAATCCAACTCACC |  |  |  |
| **M:** | **F:** AAATTAGTTTGGTTTTTATTAGGCG | 58°C | 149 |  |
|  | **R:** GATAACTAAATCGATCCGACTCG |  |  |  |
| ***NDR2/STK38L* (Genbank access : NC_000012.12)** | | | | |
| **U:** | **F:** TATTTTAGTTTGGGTGATAGAGTGA | 60°C | 123 | MethPrimer |
|  | **R:** TTAATAAAAACAAAACCACCTCAAC |  |  |  |
| **M:** | **F:** GTATTTTAGTTTGGGCGATAGAGC | 58°C | 122 |  |
|  | **R:** TAATAAAAACGAAACCACCTCGA |  |  |  |
| ***RASSF1* (Genbank access : NC_000003.12)** | | | | |
| **U:** | **F:** TTTGGTTGGAGTGTGTTAATGTG | 60°C | 108 | [60] |
|  | **R:** CAAACCCCACAAACTAAAAACAA |  |  |  |
| **M:** | **F:** GTGTTAACGCGTTGCGTATC | 62°C | 96 |  |
|  | **R:** AACCCCGCGAACTAAAAACGA |  |  |  |

**Table.S4.** Antibodies used in this work.

| Target, clone | Host | clonality | Suppliers, reference |
| --- | --- | --- | --- |
| RASSF1A | Mouse | monoclonal | Ebioscience, 14-688-82 |
| Phospho- LATS (S989) | Rabbit |  | Cell Signaling Technology 91575S |
| LATS1 | Rabbit | polyclonal | Cell Signaling Technology 9153S |
| LATS2 | Rabbit |  | Cell Signaling Technology 5888S |
| Phospho-MST1 (T183) / MST2 (T180) | Rabbit |  | Cell Signaling Technology 3681S |
| MST1 | Rabbit |  | Cell Signaling Technology 3682S |
| MST2 | Rabbit |  | Cell Signaling Technology 3952S |
| NDR1 (YJ-7) | Mouse | Monoclonal | Santa Cruz Biotechnology FO316, sc-100404 |
| NDR2 (STK38L) | Mouse | monoclonal | Lifespan Biosciences STK38L, LS-C174201-100 |
| PhosphoTyr357YAP | Rabbit |  | Cell Signaling Technology |
| PhosphoSer127YAP | Rabbit |  | Cell Signaling Technology 4911S |
| YAP total (D8H1X) | Rabbit | Monoclonal | Cell Signaling Technology 14074S |
| PhosphoSer89Taz | Rabbit |  | Cell Signaling Technology 59971 |
| Taz total (E8E9G) | Rabbit |  | Cell Signaling Technology 83669S |
| Actine | Rabbit |  | Cell Signaling Technology, 4970S |
| Tubuline | Mouse | monoclonal | Sigma 038M4837V |
| HIF1a (D1S7W) | Rabbit | monoclonal | Cell Signaling Technology 36169S |
| cadherin-N | Mouse | monoclonal | Ebioscience, 14-3259-82 |
| cadherin-E (24E10) | Rabbit | monoclonal | Cell Signaling Technology 3195S |
| Carbonic anhydrase IX (CAIX) | Rabbit | Polyclonal | Genetex, GTX15086 |
| Connexine 43 | Rabbit | Polyclonal | Cell Signaling Technology 3512S |
| Fascine (55k_2) | Mouse | monoclonal | Cell Signaling Technology 54545S |
| Phospho-C-Jun (ser73) | Rabbit | Monoclonal | Cell Signaling Technology 3270S |
| Anticorps secondaire anti-souris Ig G (HRP-linked) | Goat |  | Cell Signaling Technology 7076S |
| Anticorps secondaire anti-lapin Ig G (HRP linked) | Goat |  | Cell Signaling Technology 7074S |

**References**

56. Bordji K, Grandval A, Cuhna-Alves L, Lechapt-Zalcman E & Bernaudin M. Hypoxia-inducible factor-2α (HIF-2α), but not HIF-1α, is essential for hypoxic induction of class III β-tubulin expression in human glioblastoma cells. FEBS J 281, 5220-5236 (2014)

57. Kessler J, Hahnel A, Wichmann H, Rot S, Kappler M, Bache M et al. HIF-1α inhibition by siRNA or chetomin in human malignant glioma cells: effects on hypoxic radioresistance and monitoring via CA9 expression. BMC Cancer 10, 605 (2010)

58. He C, Sun XP, Qiao H, Jiang X, Wang D, Jin X et al. Downregulating hypoxia-inducible factor-2α improves the efficacy of doxorubicin in the treatment of hepatocellular carcinoma. Cancer Sci 103, 528-534 (2012)

59. Seidel C, Schagdarsurengin U, Blümke K, Würl P, Pfeifer GP, Hauptmann S et al. Frequent hypermethylation of MST1 and MST2 in soft tissue sarcoma. Mol Carcinog 46, 865-871 (2007)

60. Schagdarsurengin U, Gimm O, Hoang-Vu C, Dralle H, Pfeifer GP, Dammann R. Frequent epigenetic silencing of the CpG island promoter of RASSF1A in thyroid carcinoma. Cancer Res. 62, 3698-3701 (2002)
