# Supplementary material for: Hypoxia-induced activation of NDR2 underlies brain metastases from Non-Small Cell Lung Cancer
Source: Cell Death Dis. 2023 Dec 13;14(12):823. doi: 10.1038/s41419-023-06345-3 (PMC10719310; doi:10.1038/s41419-023-06345-3)
Supplement: Supplementary file 3 — Original Western Blot [file 41419_2023_6345_MOESM3_ESM.pptx]

## Slide 1
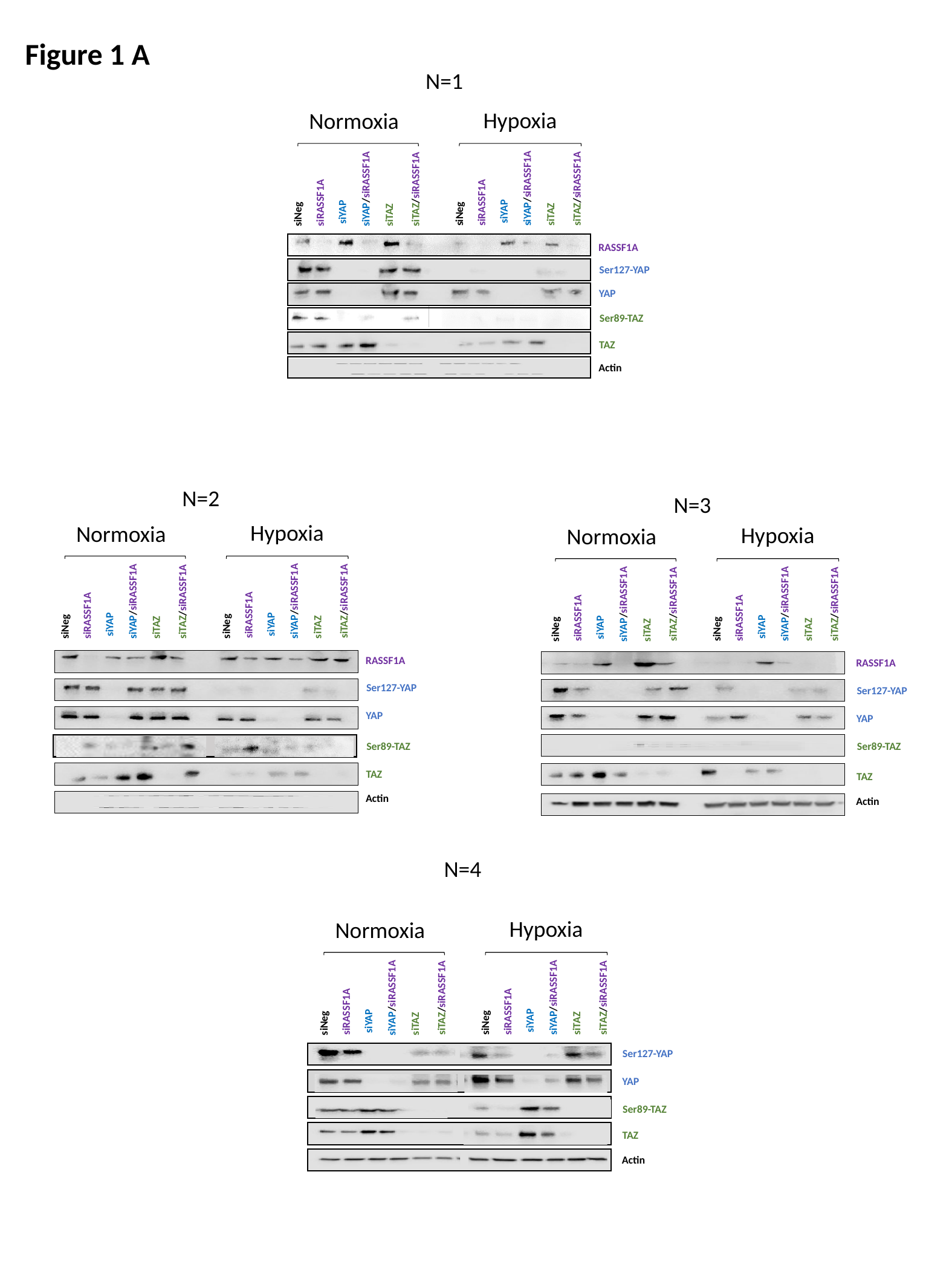

Figure 1 A
N=1
Hypoxia
Normoxia
siYAP/siRASSF1A
siTAZ/siRASSF1A
siYAP/siRASSF1A
siTAZ/siRASSF1A
siRASSF1A
siRASSF1A
 siYAP
 siYAP
siNeg
siNeg
siTAZ
siTAZ
RASSF1A
Ser127-YAP
YAP
Ser89-TAZ
TAZ
Actin
N=2
N=3
Hypoxia
Normoxia
Hypoxia
Normoxia
siYAP/siRASSF1A
siTAZ/siRASSF1A
siYAP/siRASSF1A
siTAZ/siRASSF1A
siYAP/siRASSF1A
siTAZ/siRASSF1A
siYAP/siRASSF1A
siTAZ/siRASSF1A
siRASSF1A
siRASSF1A
siRASSF1A
siRASSF1A
 siYAP
 siYAP
siNeg
siNeg
siTAZ
siTAZ
 siYAP
 siYAP
siNeg
siNeg
siTAZ
siTAZ
RASSF1A
RASSF1A
Ser127-YAP
Ser127-YAP
YAP
YAP
Ser89-TAZ
Ser89-TAZ
TAZ
TAZ
Actin
Actin
N=4
Hypoxia
Normoxia
siYAP/siRASSF1A
siTAZ/siRASSF1A
siYAP/siRASSF1A
siTAZ/siRASSF1A
siRASSF1A
siRASSF1A
 siYAP
 siYAP
siNeg
siNeg
siTAZ
siTAZ
Ser127-YAP
YAP
Ser89-TAZ
TAZ
Actin

## Slide 2
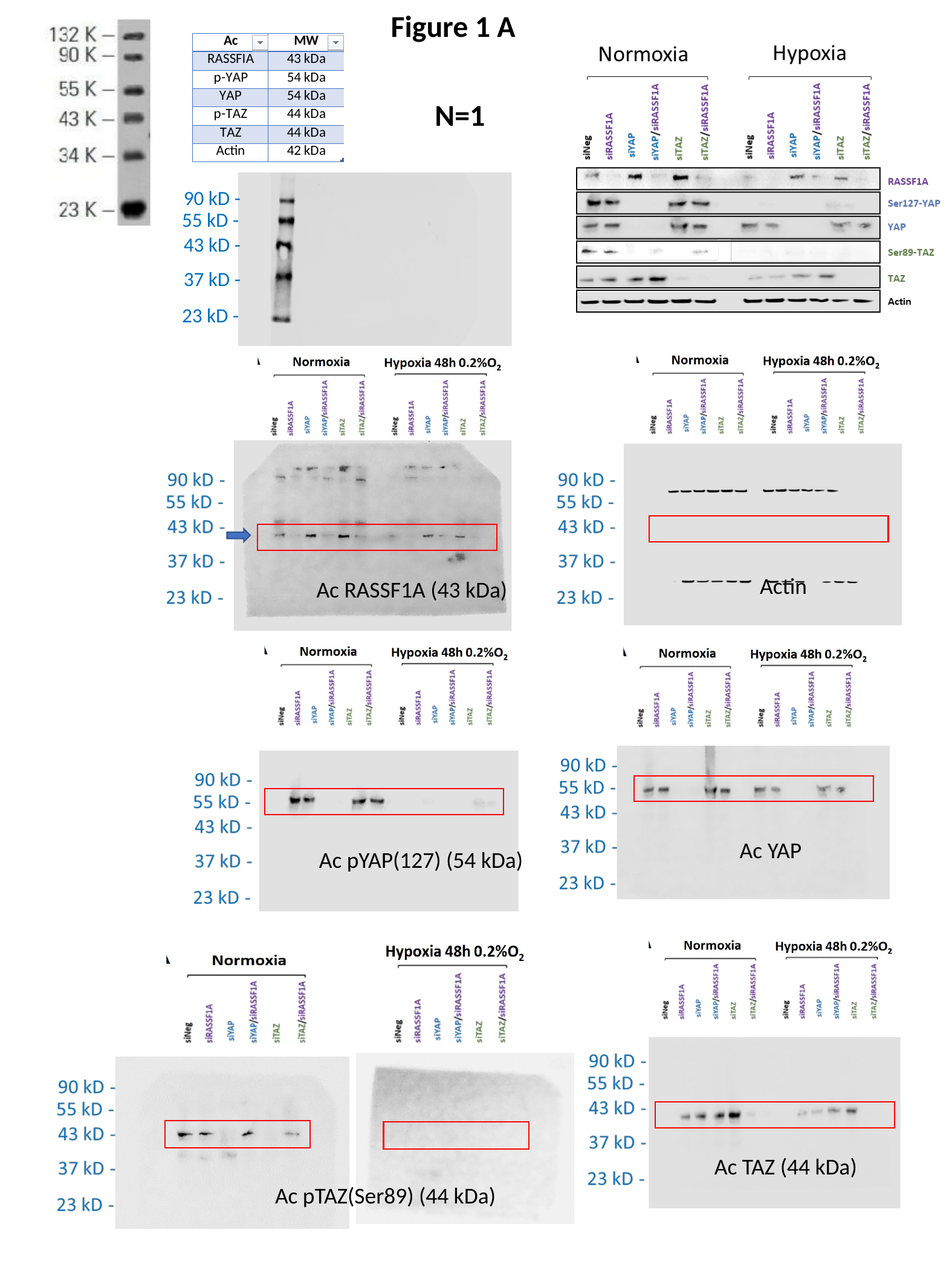

Figure 1 A
N=1
90 kD -
55 kD -
43 kD -
37 kD -
23 kD -
Actin
Ac RASSF1A (43 kDa)
Ac YAP
Ac pYAP(127) (54 kDa)
Ac TAZ (44 kDa)
Ac pTAZ(Ser89) (44 kDa)

## Slide 3
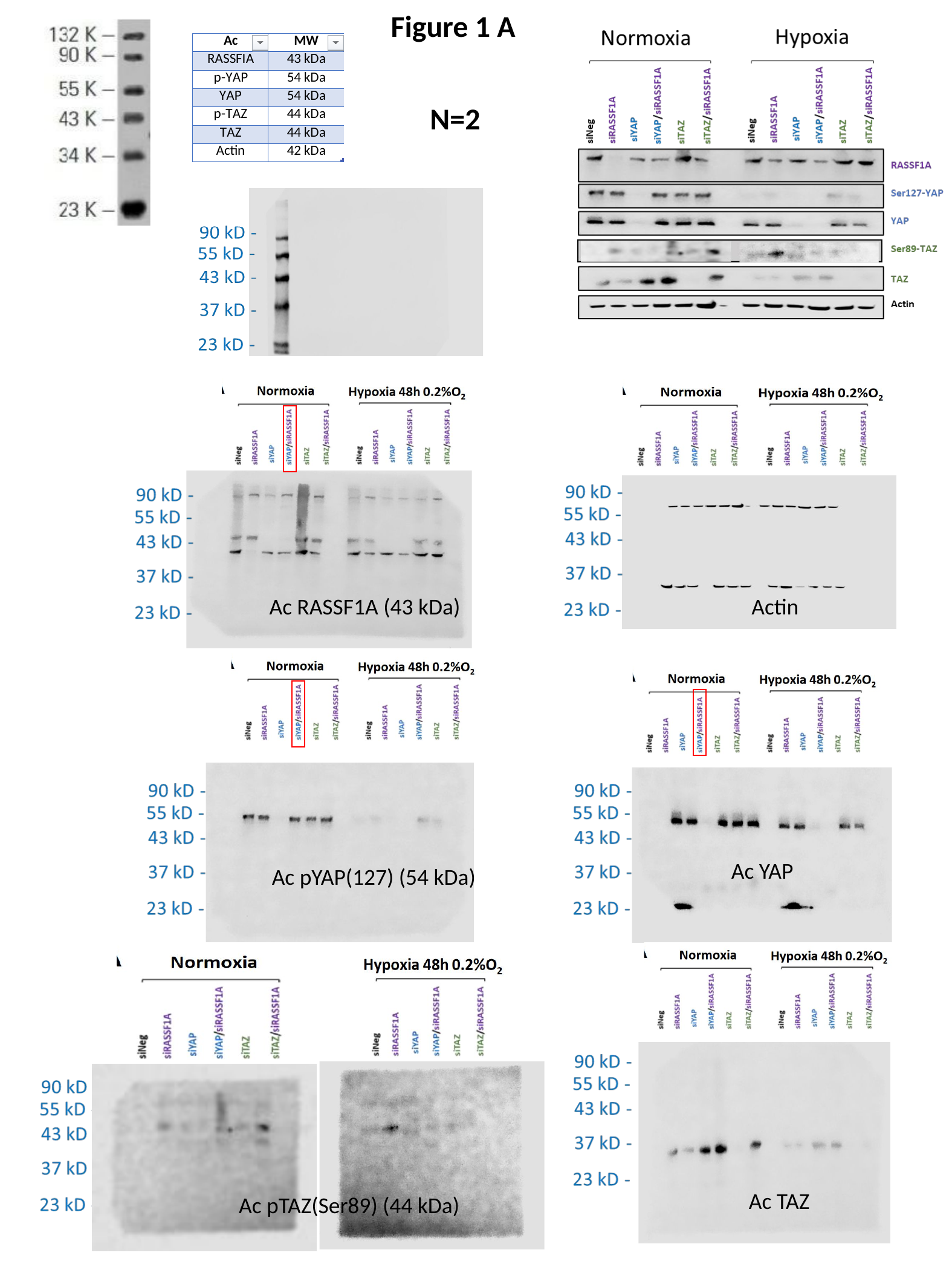

Figure 1 A
N=2
Ac RASSF1A (43 kDa)
Actin
Ac YAP
Ac pYAP(127) (54 kDa)
Ac TAZ
Ac pTAZ(Ser89) (44 kDa)

## Slide 4
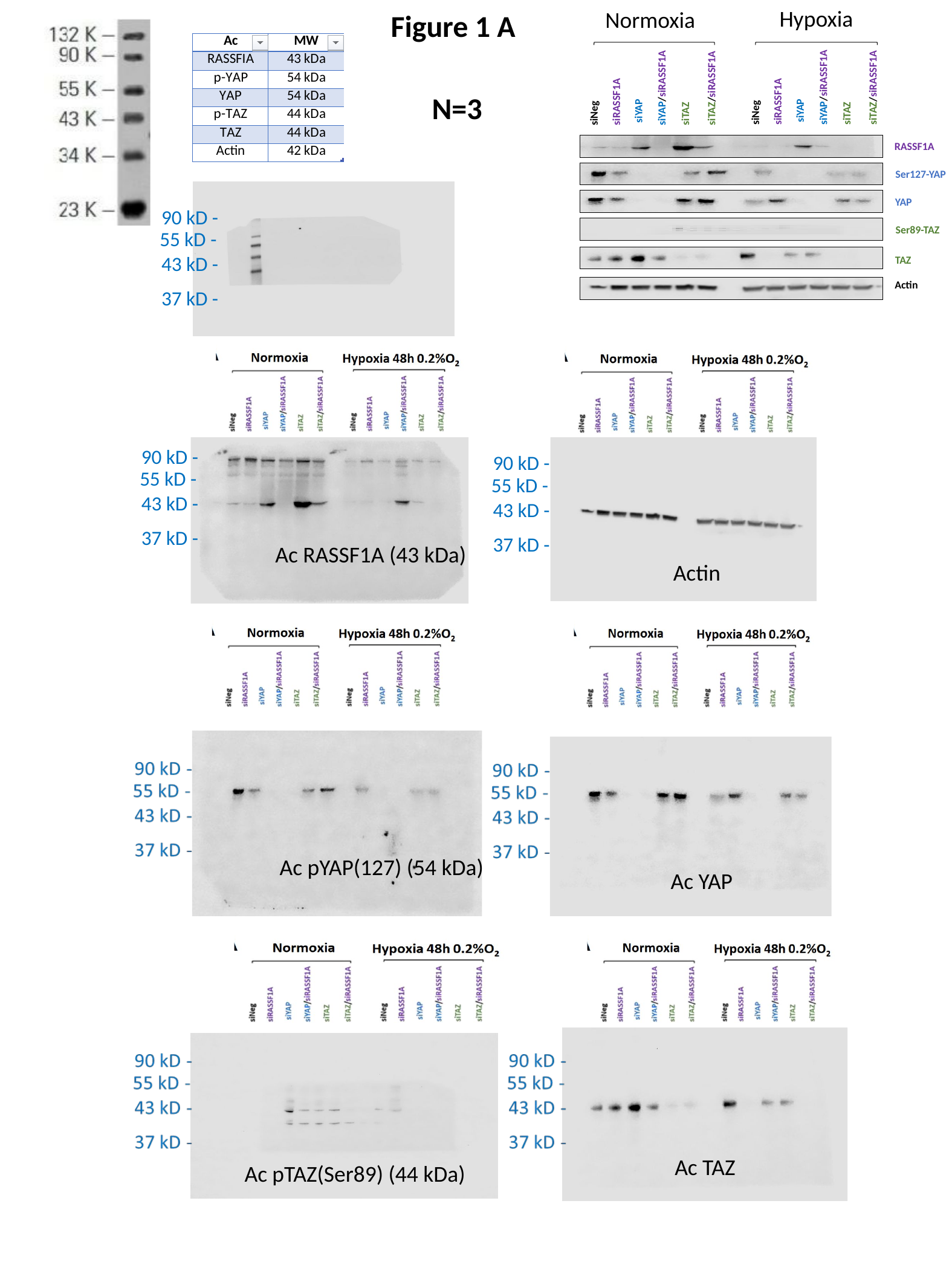

Hypoxia
Normoxia
Figure 1 A
siYAP/siRASSF1A
siTAZ/siRASSF1A
siYAP/siRASSF1A
siTAZ/siRASSF1A
N=3
siRASSF1A
siRASSF1A
 siYAP
 siYAP
siNeg
siNeg
siTAZ
siTAZ
RASSF1A
Ser127-YAP
YAP
90 kD -
Ser89-TAZ
55 kD -
43 kD -
TAZ
Actin
37 kD -
90 kD -
90 kD -
55 kD -
55 kD -
43 kD -
43 kD -
37 kD -
37 kD -
Ac RASSF1A (43 kDa)
Actin
Ac pYAP(127) (54 kDa)
Ac YAP
Ac TAZ
Ac pTAZ(Ser89) (44 kDa)

## Slide 5
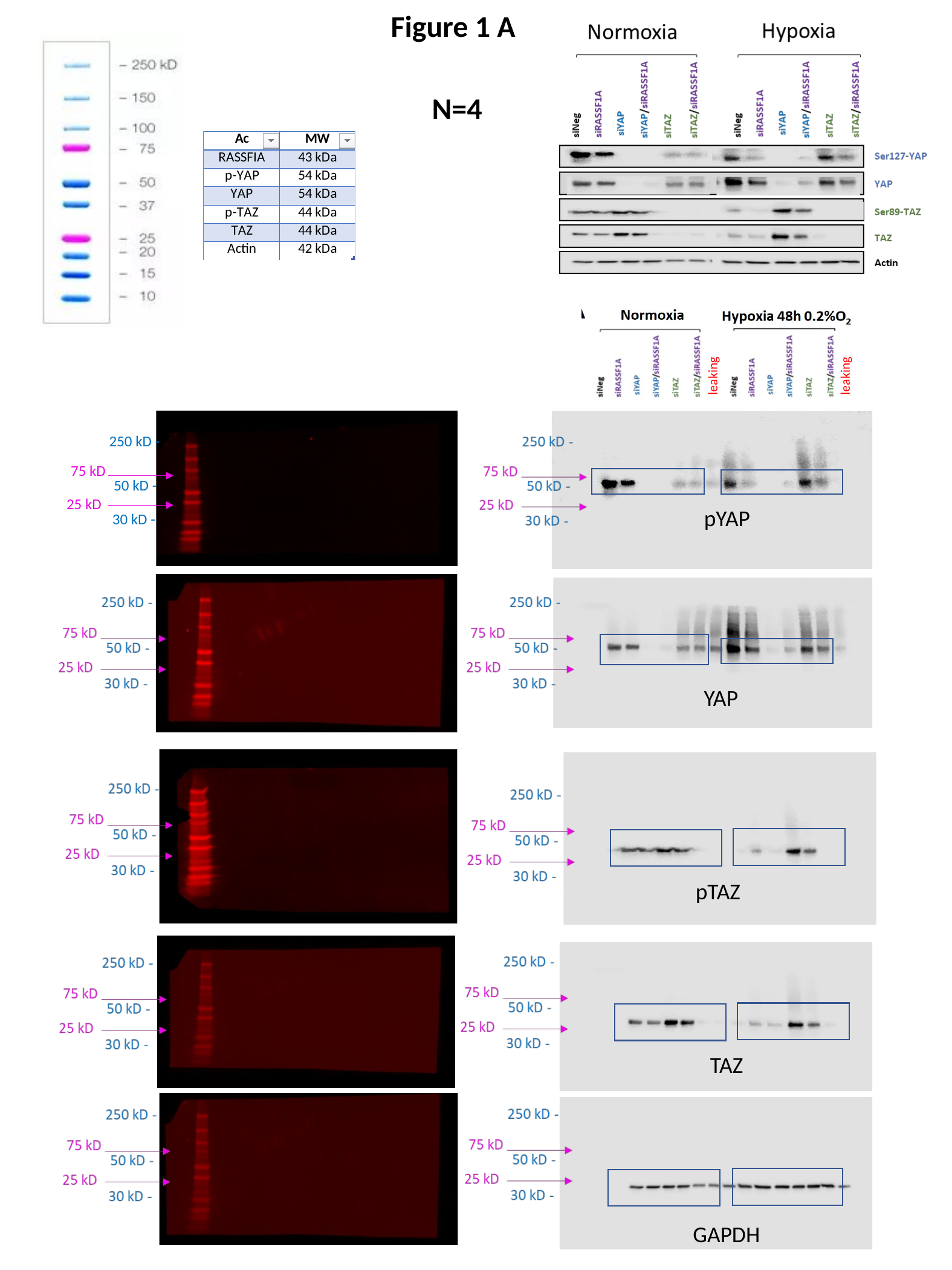

Figure 1 A
N=4
leaking
leaking
250 kD -
75 kD
50 kD -
25 kD
pYAP
30 kD -
YAP
pTAZ
TAZ
GAPDH

## Slide 6
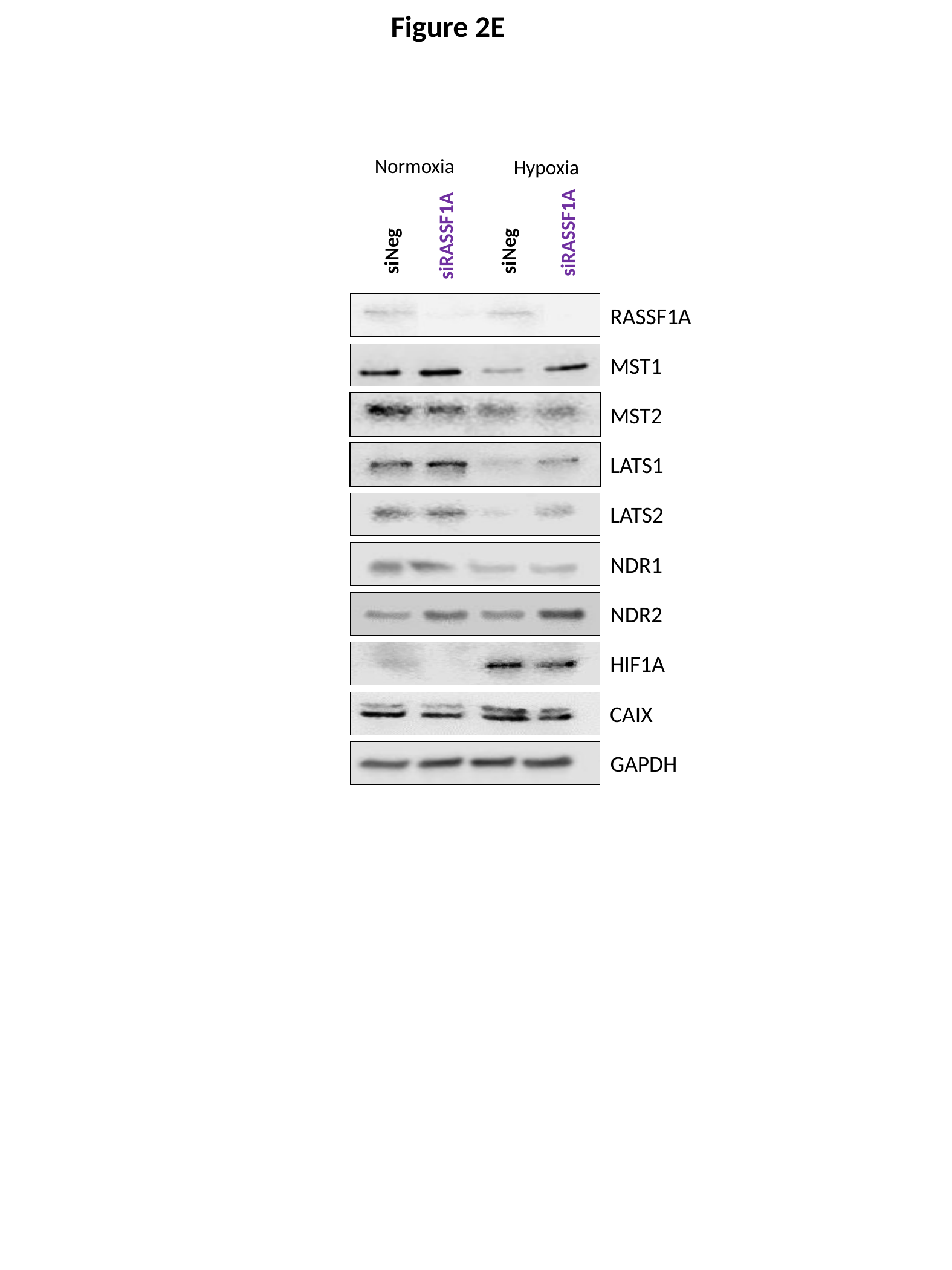

Figure 2E
Normoxia
Hypoxia
siRASSF1A
siRASSF1A
siNeg
siNeg
RASSF1A
MST1
MST2
LATS1
LATS2
NDR1
NDR2
HIF1A
CAIX
GAPDH

## Slide 7
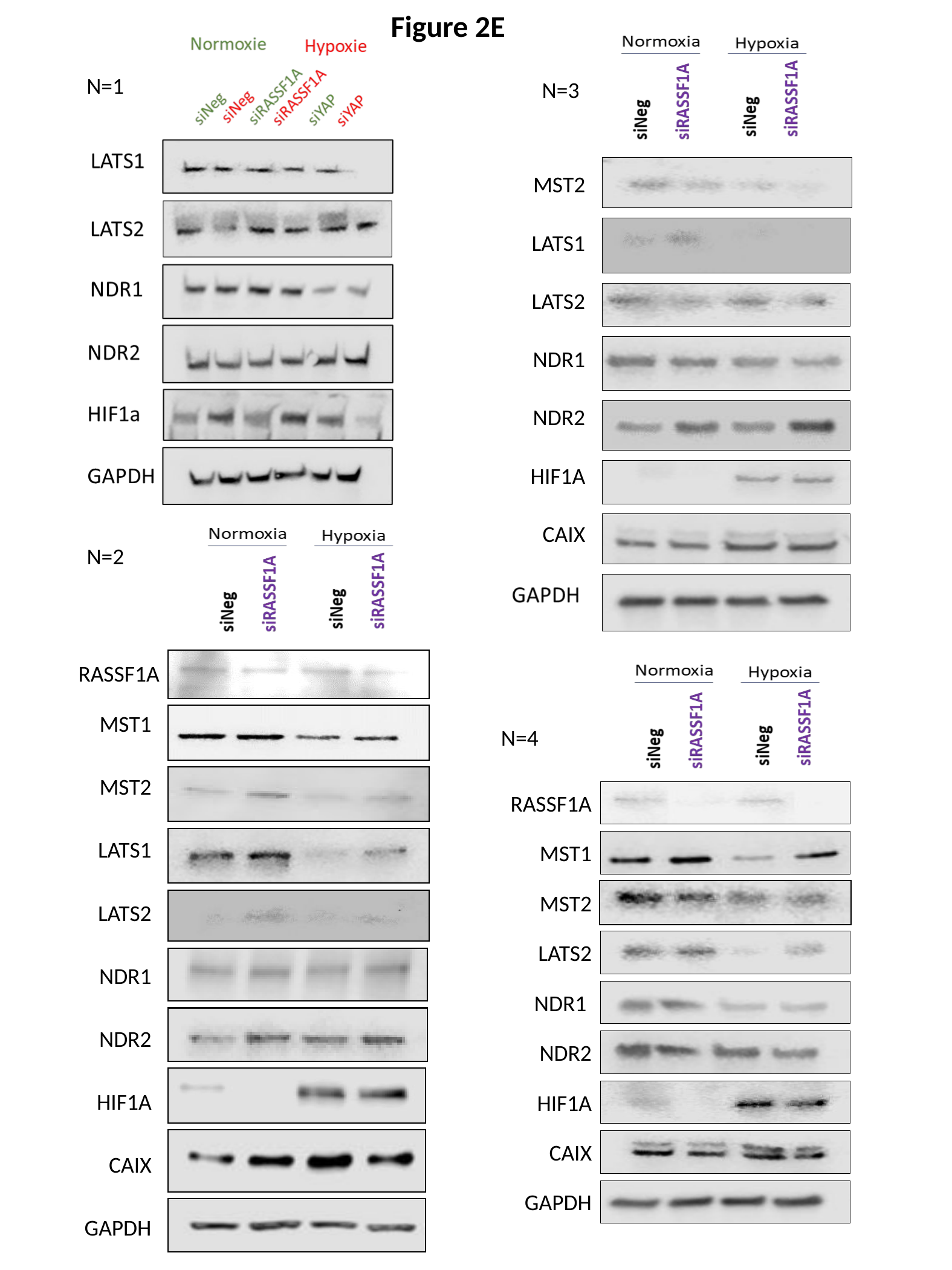

Figure 2E
N=1
N=3
MST2
LATS1
LATS2
NDR1
NDR2
HIF1A
CAIX
N=2
RASSF1A
MST1
MST2
LATS1
LATS2
NDR1
NDR2
HIF1A
CAIX
GAPDH
N=4
RASSF1A
MST1
MST2
LATS2
NDR1
NDR2
HIF1A
CAIX
GAPDH

## Slide 8
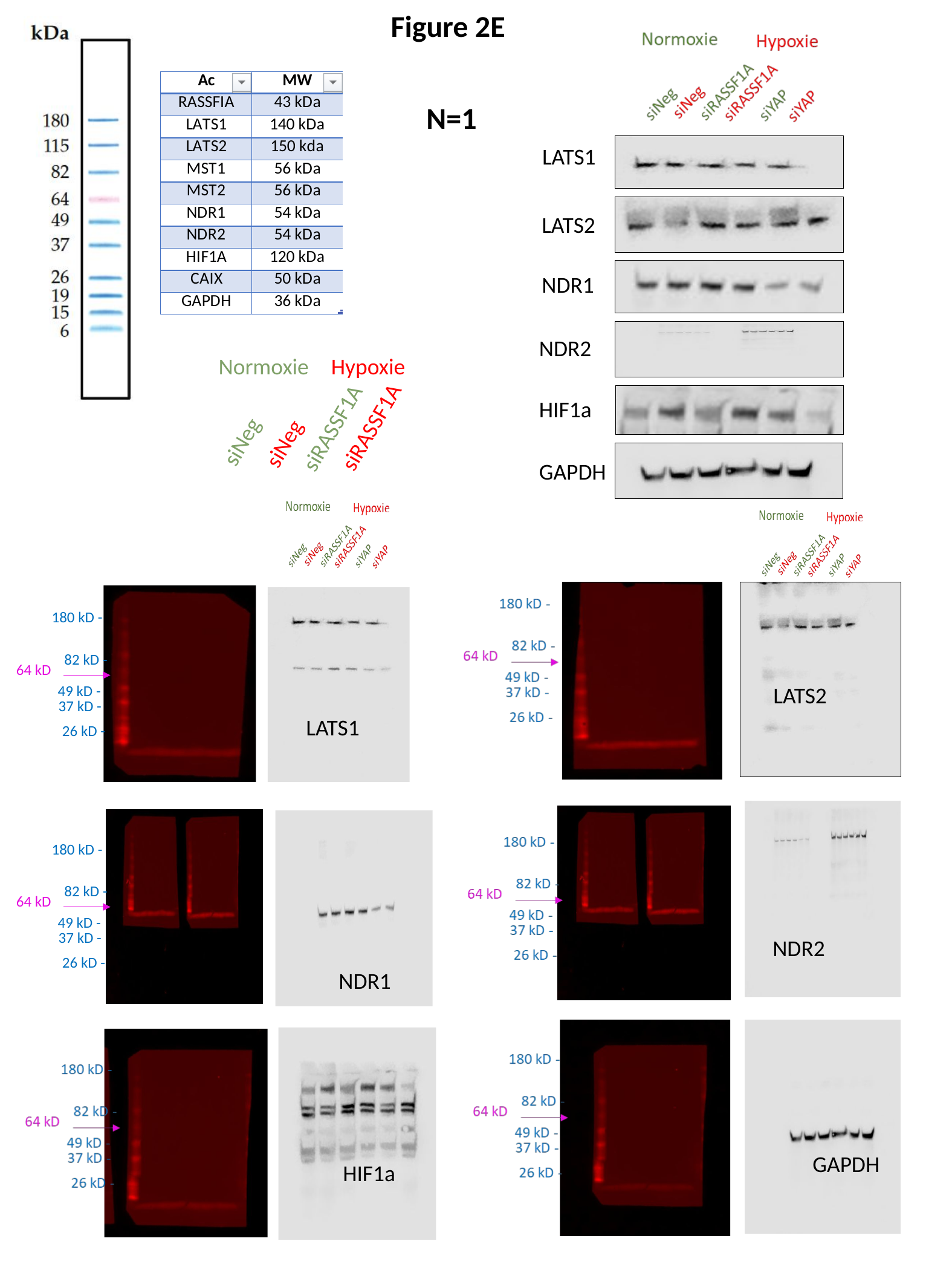

Figure 2E
N=1
LATS1
LATS2
NDR1
NDR2
Normoxie
Hypoxie
HIF1a
siRASSF1A
siRASSF1A
siNeg
siNeg
GAPDH
180 kD -
82 kD -
64 kD
LATS2
49 kD -
37 kD -
LATS1
26 kD -
180 kD -
82 kD -
64 kD
49 kD -
37 kD -
NDR2
26 kD -
NDR1
GAPDH
HIF1a

## Slide 9
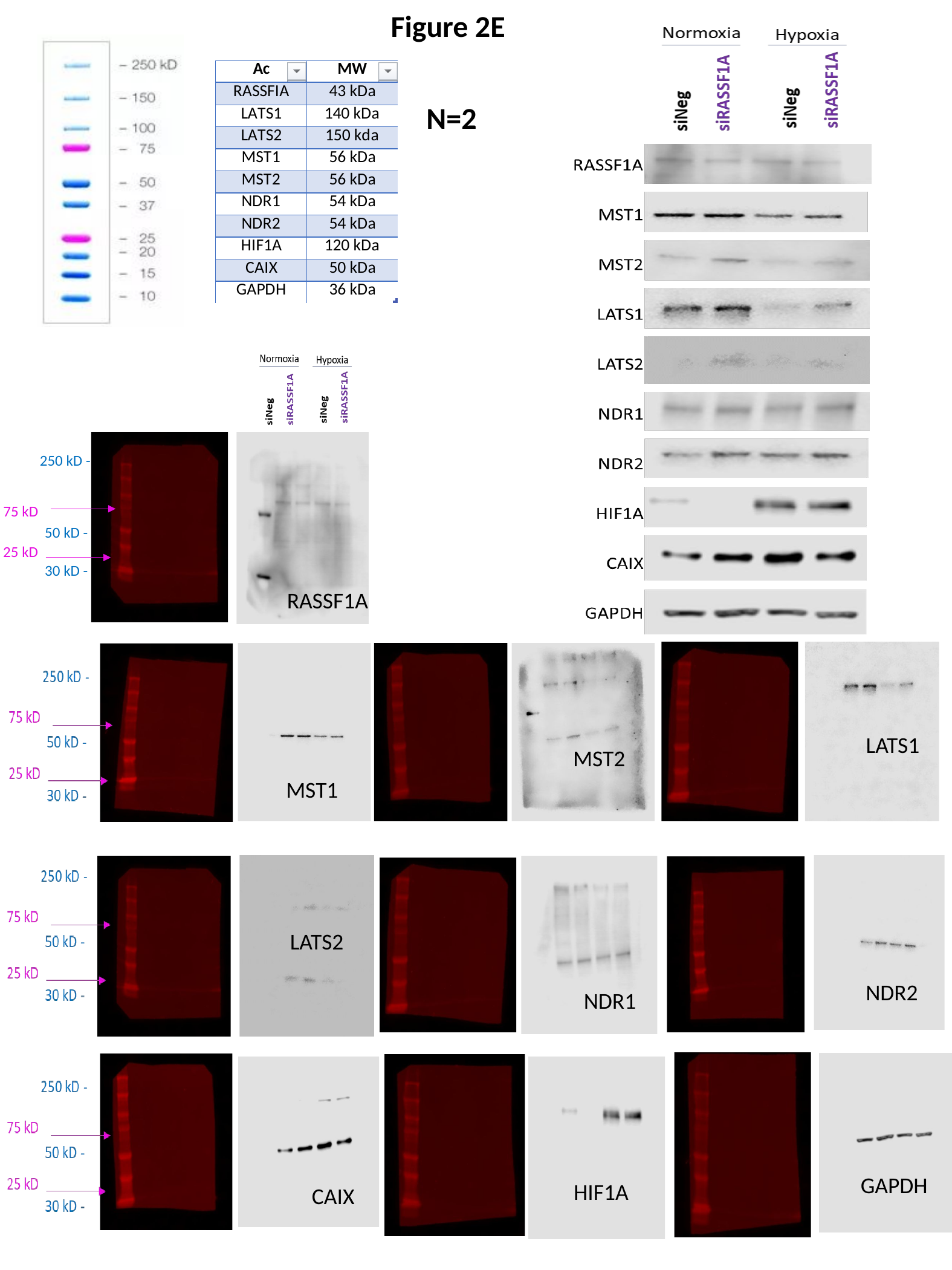

Figure 2E
N=2
250 kD -
75 kD
50 kD -
25 kD
30 kD -
RASSF1A
LATS1
MST2
MST1
LATS2
NDR2
NDR1
GAPDH
HIF1A
CAIX

## Slide 10
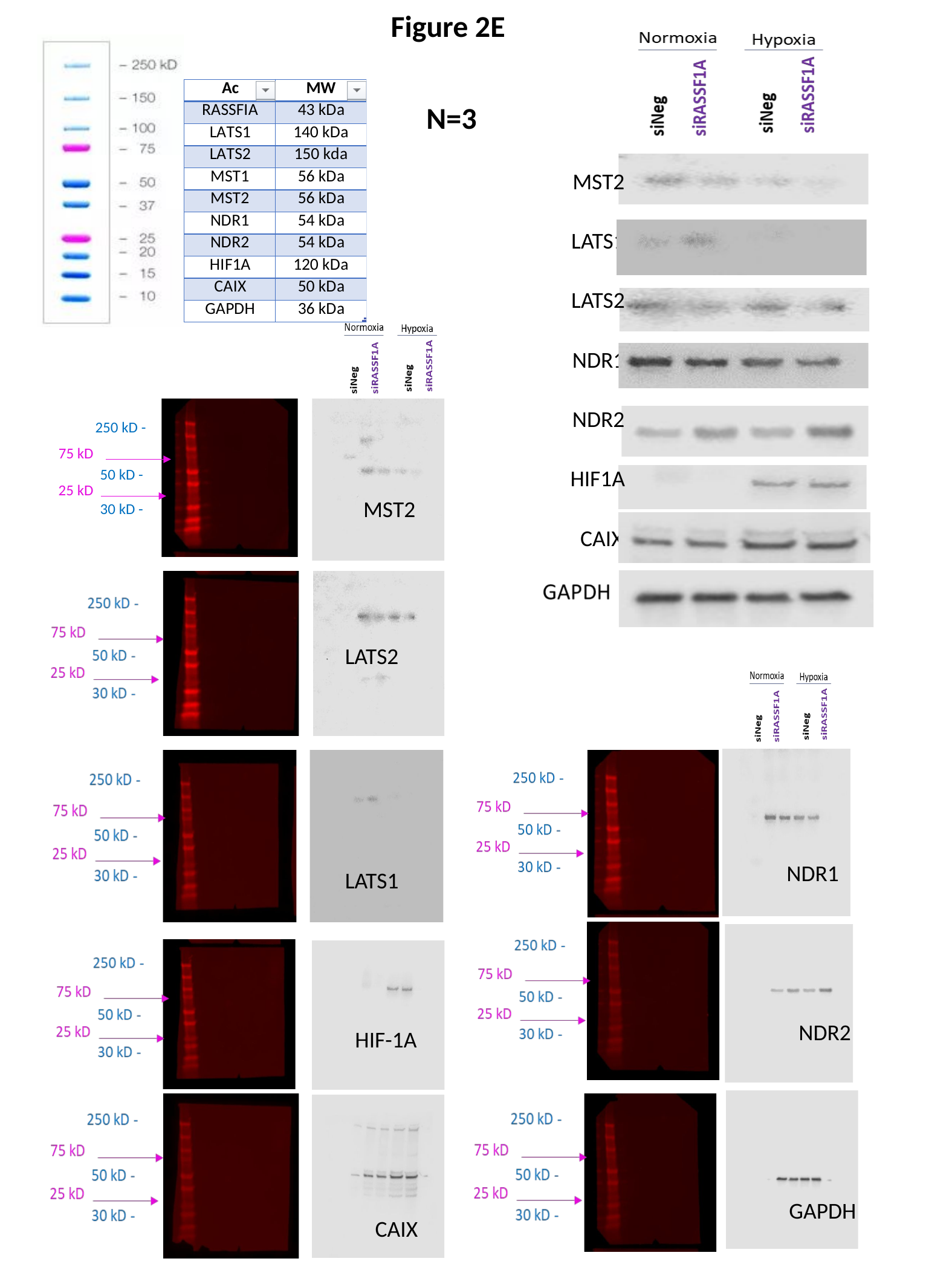

Figure 2E
N=3
MST2
LATS1
LATS2
NDR1
NDR2
250 kD -
75 kD
HIF1A
50 kD -
25 kD
MST2
30 kD -
CAIX
LATS2
NDR1
LATS1
NDR2
HIF-1A
GAPDH
CAIX

## Slide 11
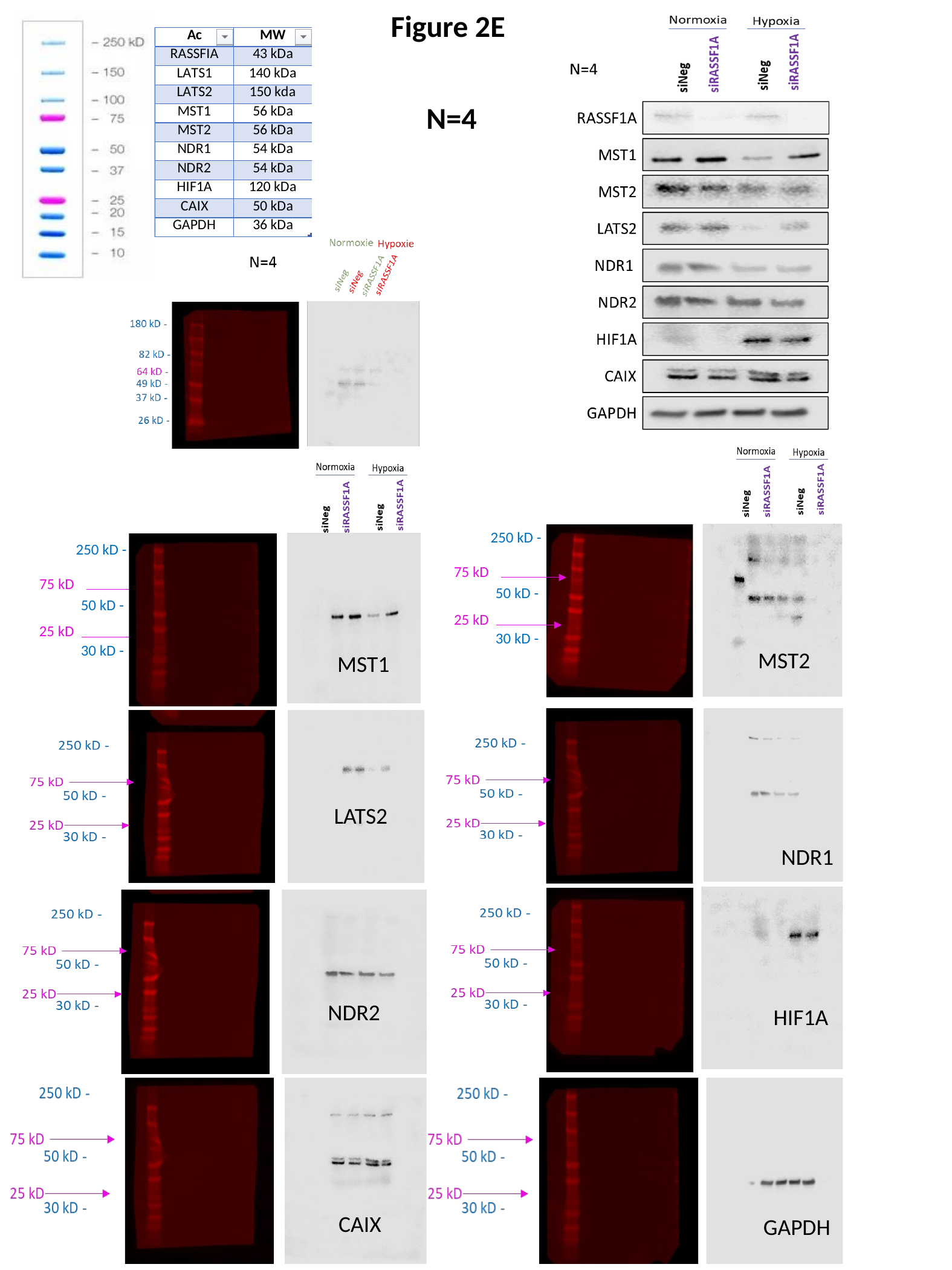

Figure 2E
N=4
250 kD -
250 kD -
75 kD
75 kD
50 kD -
50 kD -
25 kD
25 kD
30 kD -
30 kD -
MST2
MST1
LATS2
NDR1
NDR2
HIF1A
CAIX
GAPDH

## Slide 12
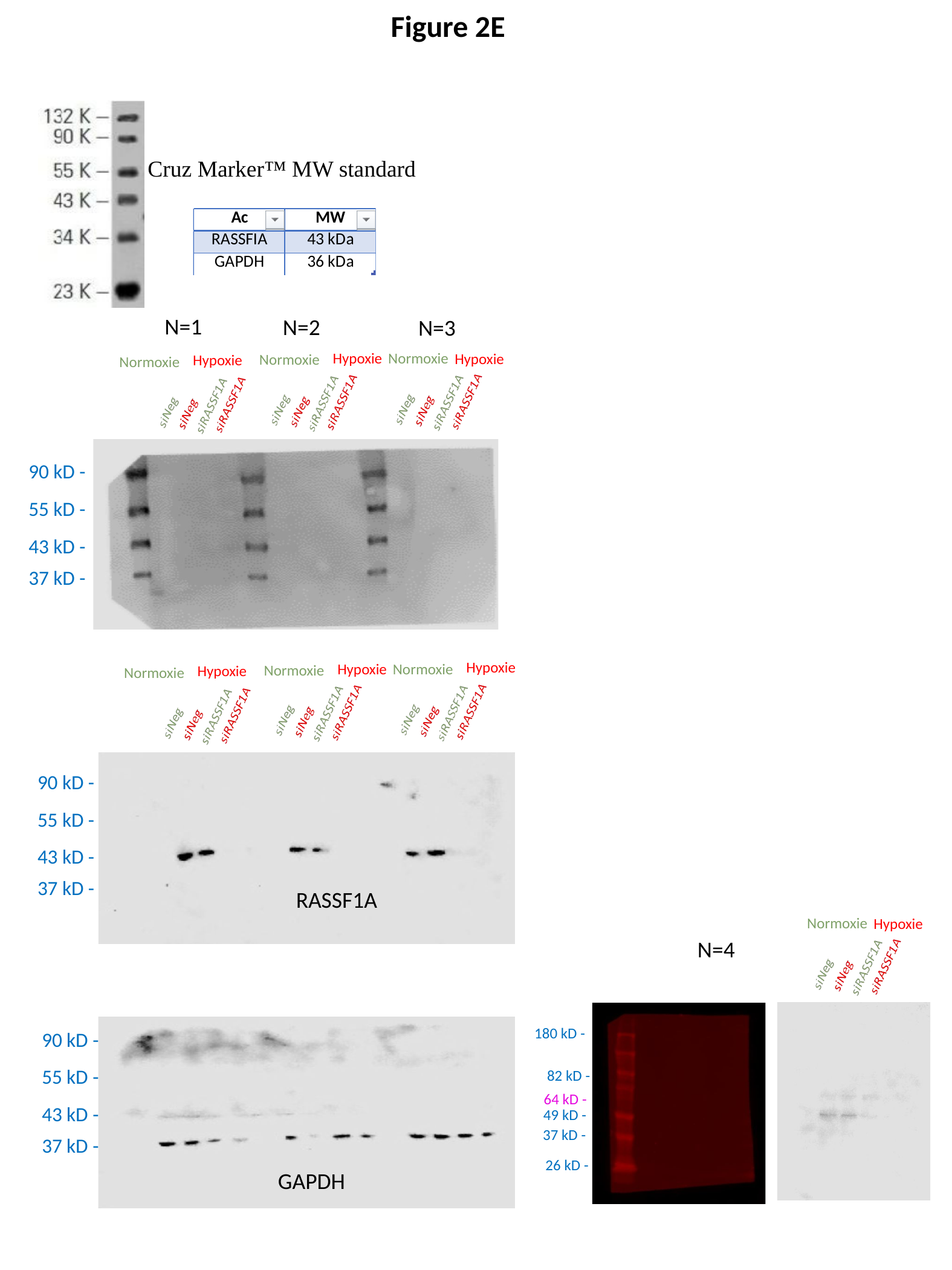

Figure 2E
Cruz Marker™ MW standard
N=1
N=2
N=3
Hypoxie
Normoxie
Hypoxie
Normoxie
Hypoxie
Normoxie
90 kD -
55 kD -
43 kD -
37 kD -
Hypoxie
Hypoxie
Normoxie
Normoxie
Hypoxie
Normoxie
90 kD -
55 kD -
43 kD -
37 kD -
RASSF1A
Normoxie
Hypoxie
N=4
180 kD -
90 kD -
55 kD -
82 kD -
64 kD -
43 kD -
49 kD -
37 kD -
37 kD -
26 kD -
GAPDH

## Slide 13
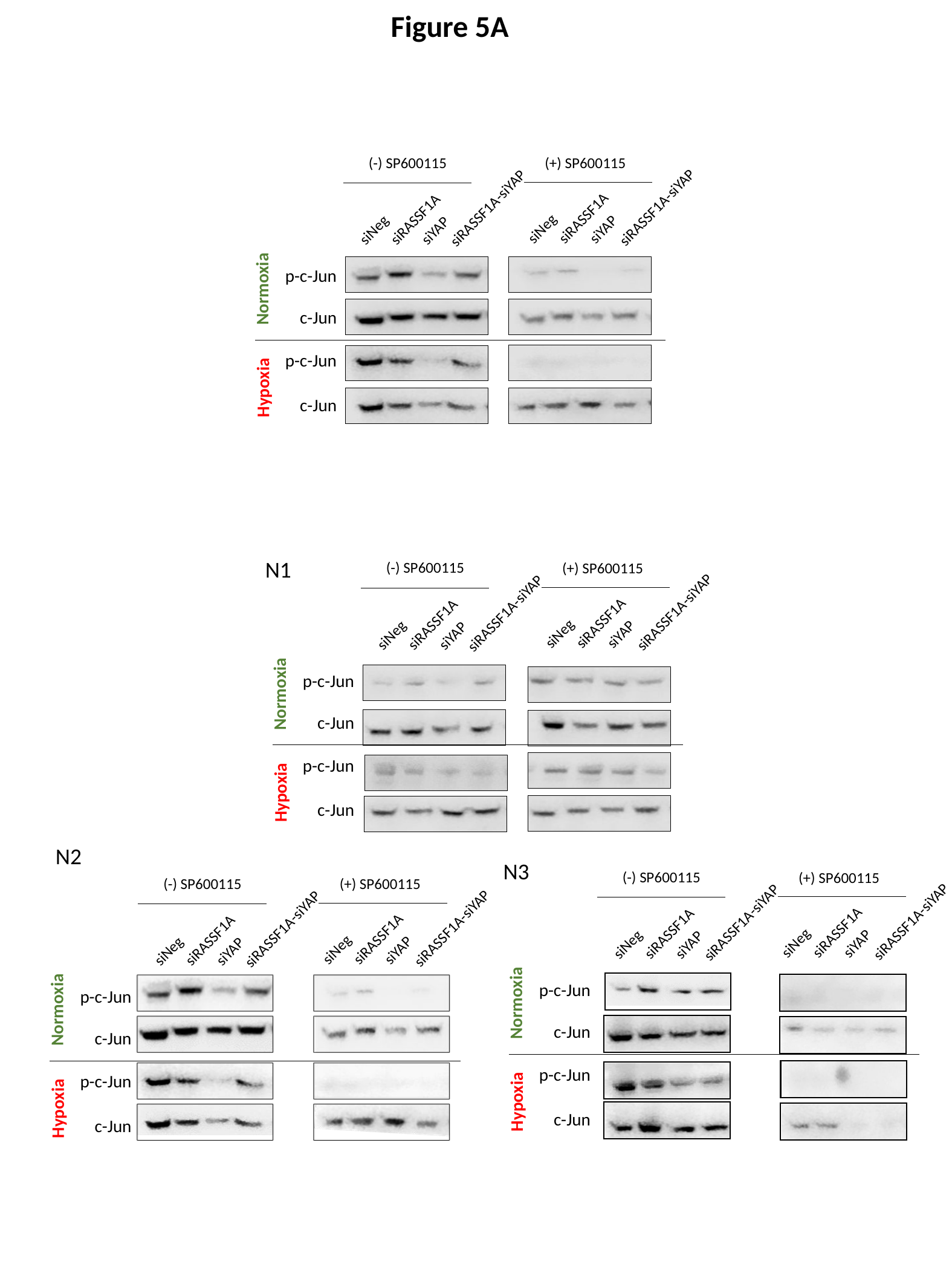

Figure 5A
(-) SP600115
(+) SP600115
siNeg
siRASSF1A
siYAP
siNeg
siRASSF1A
siYAP
siRASSF1A-siYAP
siRASSF1A-siYAP
p-c-Jun
Normoxia
c-Jun
p-c-Jun
Hypoxia
c-Jun
N1
(-) SP600115
(+) SP600115
siNeg
siRASSF1A
siYAP
siNeg
siRASSF1A
siYAP
siRASSF1A-siYAP
siRASSF1A-siYAP
p-c-Jun
Normoxia
c-Jun
p-c-Jun
Hypoxia
c-Jun
N2
N3
(-) SP600115
(+) SP600115
(-) SP600115
(+) SP600115
siNeg
siRASSF1A
siYAP
siNeg
siRASSF1A
siYAP
siRASSF1A-siYAP
siRASSF1A-siYAP
siNeg
siRASSF1A
siYAP
siNeg
siRASSF1A
siYAP
siRASSF1A-siYAP
siRASSF1A-siYAP
p-c-Jun
p-c-Jun
Normoxia
Normoxia
c-Jun
c-Jun
p-c-Jun
p-c-Jun
Hypoxia
Hypoxia
c-Jun
c-Jun

## Slide 14
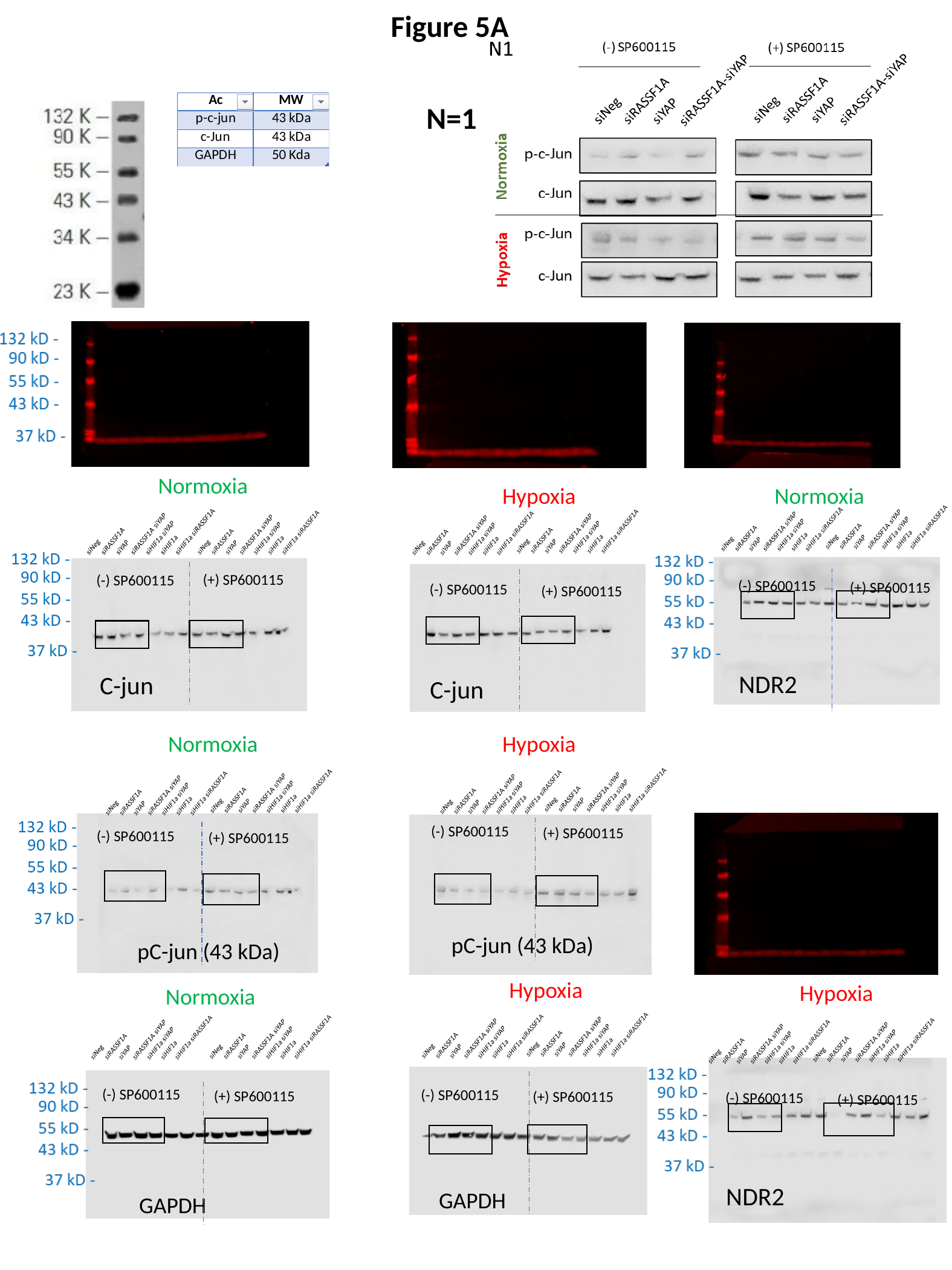

Figure 5A
N=1
Normoxia
Normoxia
Hypoxia
(+) SP600115
(-) SP600115
(-) SP600115
(+) SP600115
(-) SP600115
(+) SP600115
NDR2
C-jun
C-jun
Normoxia
Hypoxia
(-) SP600115
(+) SP600115
(-) SP600115
(+) SP600115
pC-jun (43 kDa)
pC-jun (43 kDa)
Hypoxia
Hypoxia
Normoxia
(-) SP600115
(-) SP600115
(+) SP600115
(+) SP600115
(-) SP600115
(+) SP600115
NDR2
GAPDH
GAPDH

## Slide 15
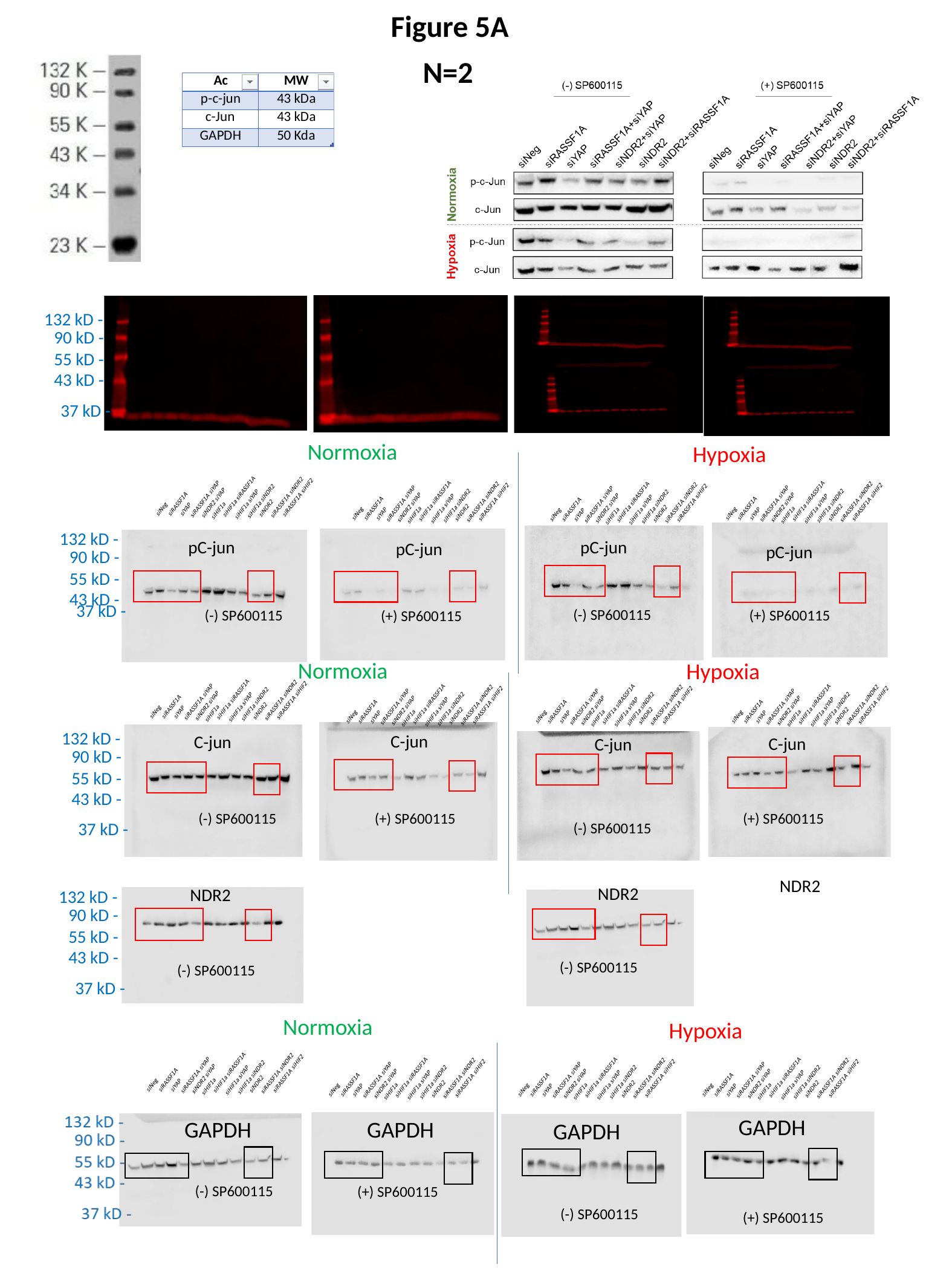

Figure 5A
N=2
132 kD -
90 kD -
55 kD -
43 kD -
37 kD -
Normoxia
Hypoxia
132 kD -
pC-jun
pC-jun
pC-jun
pC-jun
90 kD -
55 kD -
43 kD -
37 kD -
(-) SP600115
(-) SP600115
(+) SP600115
(+) SP600115
Normoxia
Hypoxia
132 kD -
C-jun
C-jun
C-jun
C-jun
90 kD -
55 kD -
43 kD -
(-) SP600115
(-) SP600115
(+) SP600115
(+) SP600115
37 kD -
(-) SP600115
NDR2
NDR2
NDR2
132 kD -
90 kD -
55 kD -
43 kD -
(-) SP600115
(-) SP600115
37 kD -
Normoxia
Hypoxia
GAPDH
GAPDH
GAPDH
GAPDH
(-) SP600115
(+) SP600115
(-) SP600115
(+) SP600115

## Slide 16
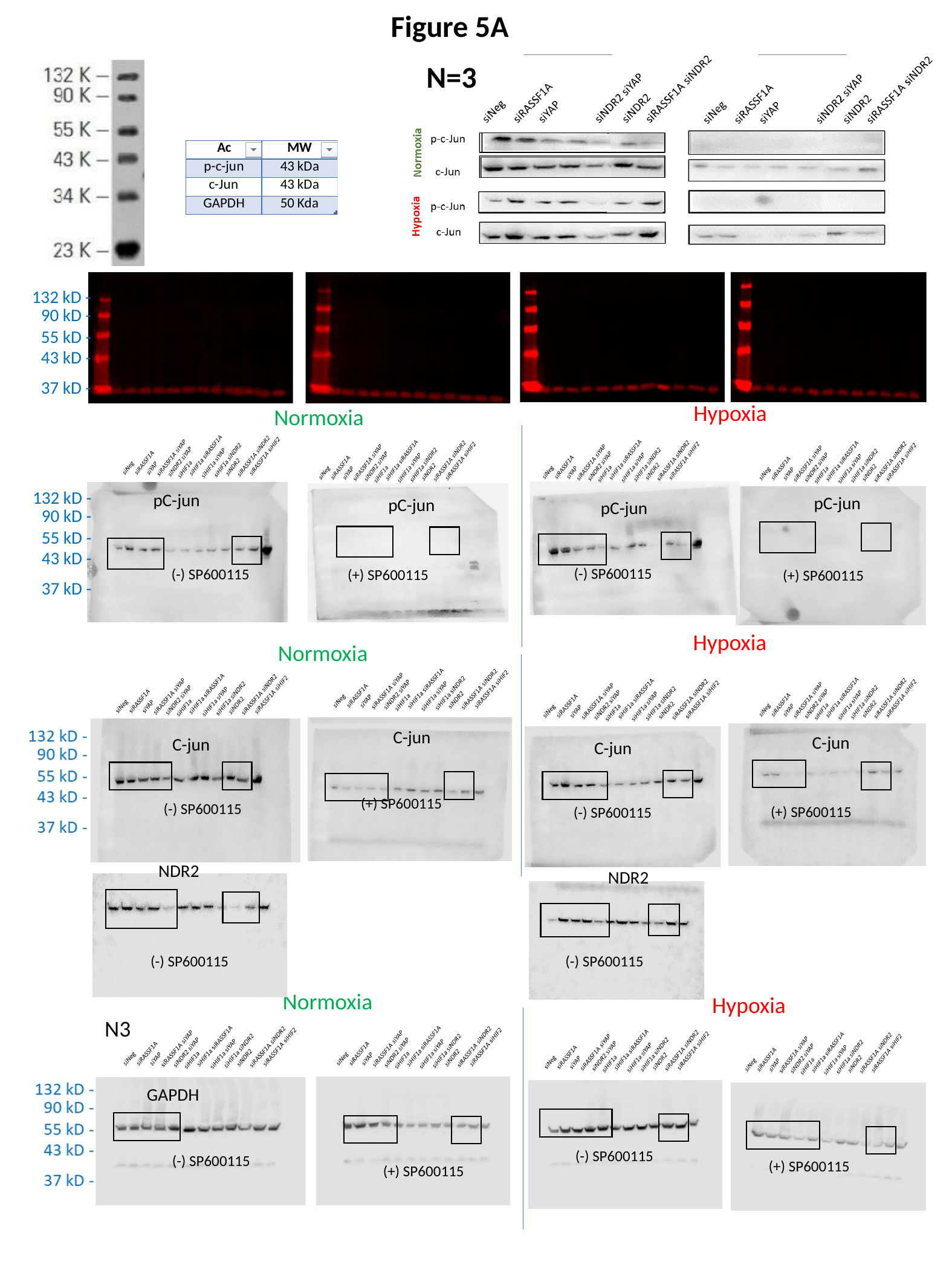

Figure 5A
N=3
132 kD -
90 kD -
55 kD -
43 kD -
37 kD -
Hypoxia
Normoxia
132 kD -
pC-jun
pC-jun
pC-jun
pC-jun
90 kD -
55 kD -
43 kD -
(-) SP600115
(-) SP600115
(+) SP600115
(+) SP600115
37 kD -
Hypoxia
Normoxia
C-jun
C-jun
C-jun
C-jun
(+) SP600115
(-) SP600115
(+) SP600115
(-) SP600115
NDR2
NDR2
(-) SP600115
(-) SP600115
Normoxia
Hypoxia
N3
GAPDH
(-) SP600115
(-) SP600115
(+) SP600115
(+) SP600115

## Slide 17
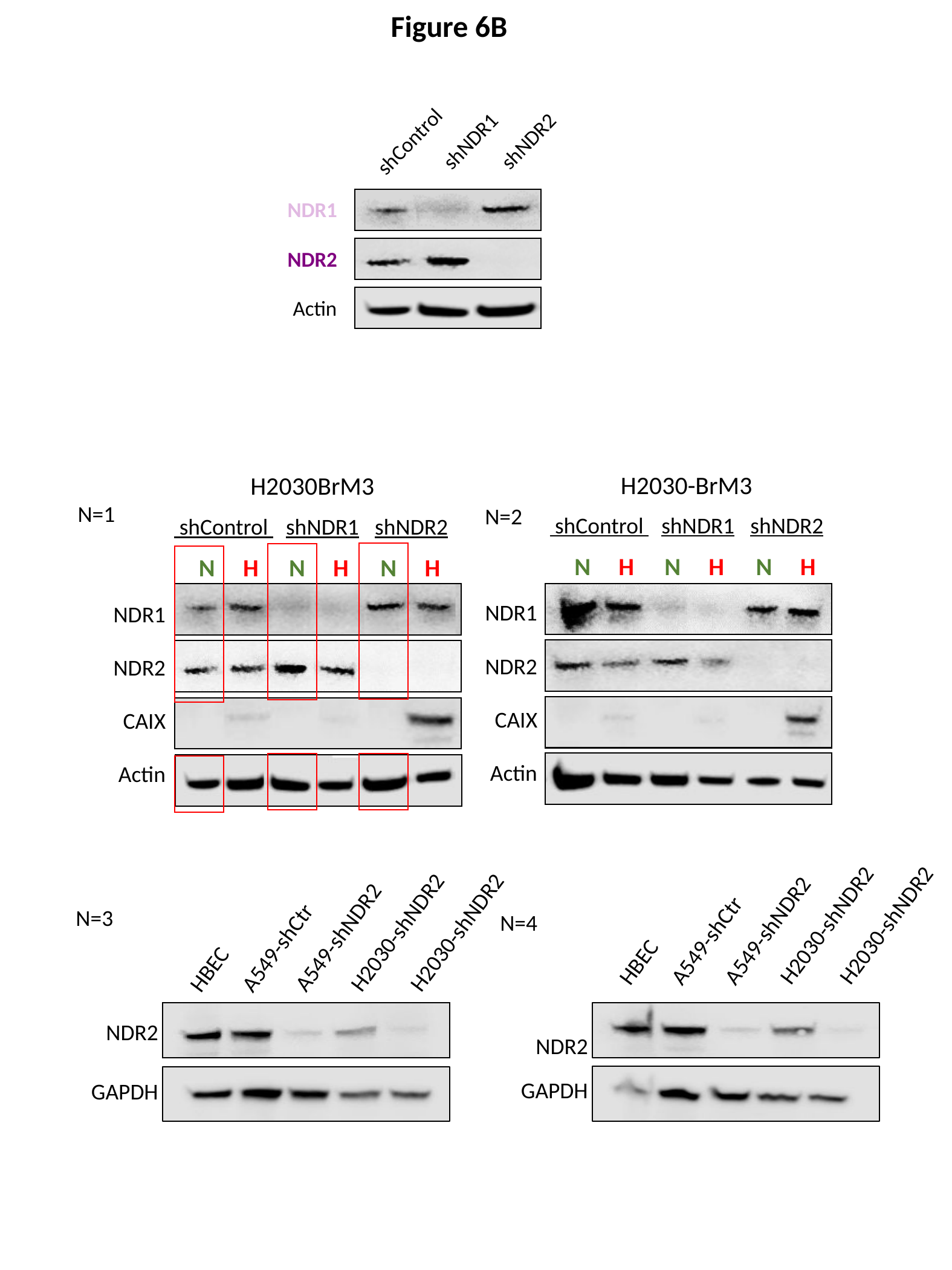

Figure 6B
 shControl
shNDR1
shNDR2
NDR1
NDR2
Actin
H2030-BrM3
H2030BrM3
N=1
N=2
 shControl
shNDR1
shNDR2
 shControl
shNDR1
shNDR2
N H
N H
N H
N H
N H
N H
NDR1
NDR1
NDR2
NDR2
CAIX
CAIX
Actin
Actin
N=3
N=4
H2030-shNDR2
H2030-shNDR2
A549-shNDR2
H2030-shNDR2
H2030-shNDR2
A549-shNDR2
A549-shCtr
A549-shCtr
HBEC
HBEC
NDR2
NDR2
GAPDH
GAPDH

## Slide 18
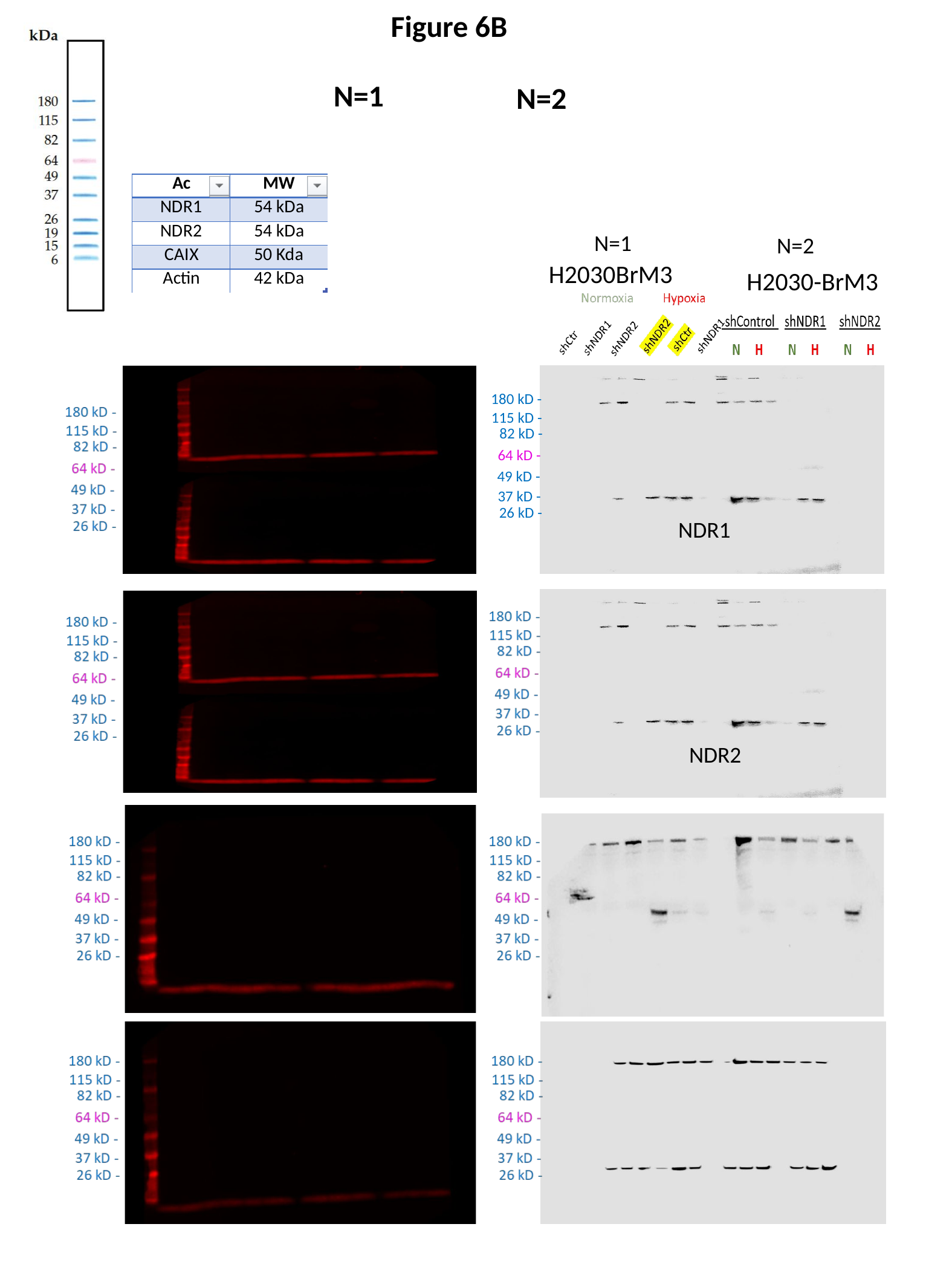

Figure 6B
N=1
N=2
N=1
N=2
H2030BrM3
H2030-BrM3
180 kD -
115 kD -
82 kD -
64 kD -
49 kD -
37 kD -
26 kD -
NDR1
NDR2

## Slide 19
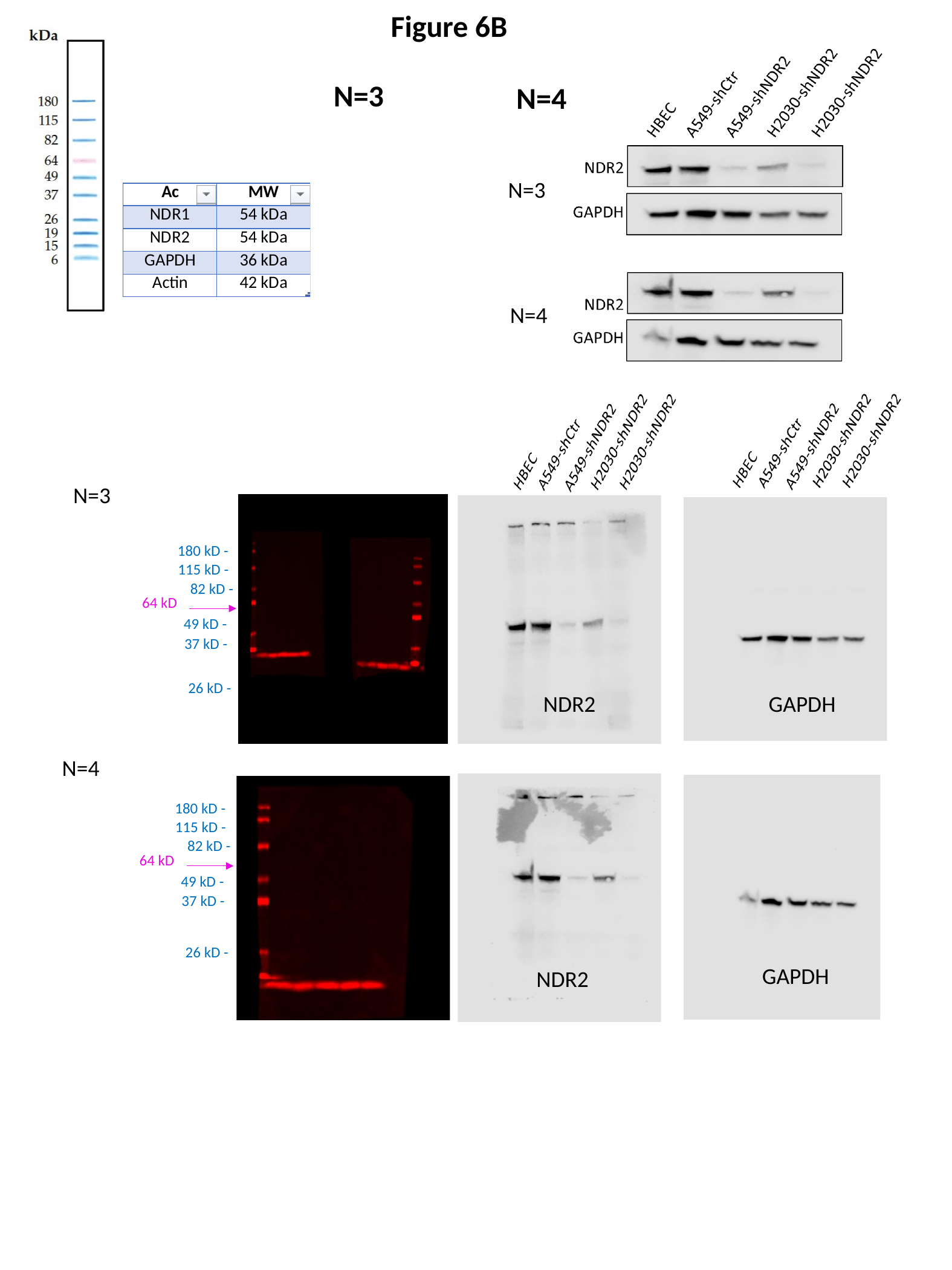

Figure 6B
N=3
N=4
N=3
N=4
N=3
180 kD -
115 kD -
82 kD -
64 kD
49 kD -
37 kD -
26 kD -
NDR2
GAPDH
N=4
180 kD -
115 kD -
82 kD -
64 kD
49 kD -
37 kD -
26 kD -
GAPDH
NDR2
